# Identification of a seven glycopeptide signature for malignant pleural mesothelioma in human serum by selected reaction monitoring

**DOI:** 10.1186/1559-0275-10-16

**Published:** 2013-11-08

**Authors:** Ferdinando Cerciello, Meena Choi, Annalisa Nicastri, Damaris Bausch-Fluck, Annemarie Ziegler, Olga Vitek, Emanuela Felley-Bosco, Rolf Stahel, Ruedi Aebersold, Bernd Wollscheid

**Affiliations:** 1 Department of Biology, Institute of Molecular Systems Biology, Swiss Federal Institute of Technology (ETH) Zürich, Wolfgang-Pauli Strasse 16, 8093 Zürich, Switzerland; 2 Laboratory of Molecular Oncology, Clinic of Oncology, University Hospital Zürich, Häldeliweg 4, 8044 Zürich, Switzerland; 3 Department of Statistics, Purdue University, West Lafayette, IN, USA; 4 Proteomics@UMG, Department of Experimental and Clinical Medicine, “Magna Græcia” University of Catanzaro, Catanzaro, Italy; 5 NCCR Neuro Center for Proteomics, University and Swiss Federal Institute of Technology (ETH) Zürich, Zürich, Switzerland; 6 Department of Computer Science, Purdue University, West Lafayette, IN, USA; 7 Faculty of Science, University Zürich, Zürich, Switzerland; 8 Department of Health Sciences and Technology, Swiss Federal Institute of Technology (ETH) Zürich, Zürich, Switzerland; 9 Present address: Centro de Genética Humana, Clínica Alemana-Universidad del Desarrollo, Santiago, Chile

**Keywords:** Malignant pleural mesothelioma, Selected reaction monitoring, Surfaceome, Targeted proteomics, Serum biomarkers

## Abstract

**Background:**

Serum biomarkers can improve diagnosis and treatment of malignant pleural mesothelioma (MPM). However, the evaluation of potential new serum biomarker candidates is hampered by a lack of assay technologies for their clinical evaluation. Here we followed a hypothesis-driven targeted proteomics strategy for the identification and clinical evaluation of MPM candidate biomarkers in serum of patient cohorts.

**Results:**

Based on the hypothesis that cell surface exposed glycoproteins are prone to be released from tumor-cells to the circulatory system, we screened the surfaceome of model cell lines for potential MPM candidate biomarkers. Selected Reaction Monitoring (SRM) assay technology allowed for the direct evaluation of the newly identified candidates in serum. Our evaluation of 51 candidate biomarkers in the context of a training and an independent validation set revealed a reproducible glycopeptide signature of MPM in serum which complemented the MPM biomarker mesothelin.

**Conclusions:**

Our study shows that SRM assay technology enables the direct clinical evaluation of protein-derived candidate biomarker panels for which clinically reliable ELISA’s currently do not exist.

## Background

Malignant pleural mesothelioma (MPM) is a fatal cancer of the pleura induced by asbestos exposure. Treatments developed over the last decade have improved patient survival [1-5]. However, their efficacy is limited by the frequent detection of MPM only at advanced stages [6,7]. Easily and longitudinally accessible blood biomarkers are expected to support diagnosis and therapy selection at early disease stages, when benefit from treatment is the highest [8]. To date, the best available MPM biomarker in serum is mesothelin [9]. While the protein is frequently elevated at advanced stages of the disease, its value for early detection remains limited [10]. The search and evaluation of additional MPM biomarkers in serum remains thus a priority. Generally, this is approached applying enzyme linked immunosorbent assays (ELISA), which commonly allow for the reliable evaluation of only one biomarker candidate at the time, like in the case of the recently proposed fibulin-3 protein [11]. An alternative would be the investigation of panels of simultaneously measured biomarkers. Such a multiplexed strategy would be a more efficient approach in terms of samples consumption and diagnostic accuracy [12]. To achieve this goal, in our study we developed and applied a hypothesis-driven targeted proteomics strategy which enabled the parallel quantitative evaluation of potential MPM candidate biomarkers in serum through SRM assay technology.

SRM assay technology relies on the ability of a triple-quadrupole mass spectrometer (QQQ) to selectively isolate predefined peptides of interest in a complex protein mixture after enzymatic digestion (usually using trypsin) [13]. SRM-assays encompass the analytical coordinates necessary for the unambiguous detection and quantification of the target candidates [14]. They consist of selected peptide-transitions to monitor (the pairs of signals representing the precursor peptide-ion and a corresponding fragment-ion), best collision energies to apply for peptide-fragmentations in the mass spectrometer and retention times of the target peptides in a chromatographic separation column. In a single SRM analysis, dozens of peptides are simultaneously quantified in complex samples with high sensitivity and reproducibility as surrogates of their proteins [15]. This multiplexing potential enables parallel testing and clinical evaluation of proposed candidate biomarkers in clinically relevant specimens [16,17].

**Figure 1:**
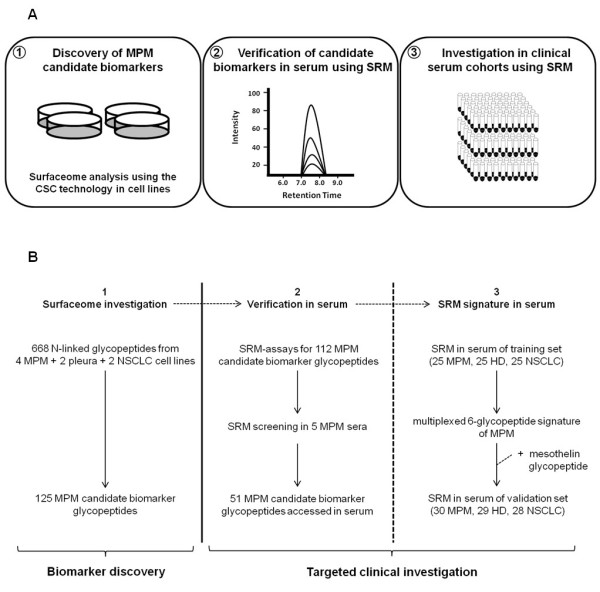
**Workflow of the study. (A)** MPM candidate biomarker glycopeptides were identified from the surfaceome of model cell lines (1). Detection in serum was targeted verified by SRM assay technology (2). Diagnostic significance was investigated by multiplexed SRM assay technology in clinical cohorts (3). **(B)** CSC technology was applied for the quantitative surfaceome screening of four MPM, two non-cancerous pleural and two NSCLC (lung adenocarcinoma) cell lines for MPM candidate biomarker N-glycopeptides (Additional file 4: Table S3) (1). SRM-assays were generated for the MPM candidate biomarkers and applied for the screening of five MPM sera (Additional file 7: Table S4 and Additional file 5: Skyline file) (2). SRM-assays for MPM candidate biomarkers detected in serum were multiplexed in one single SRM-method and quantitatively evaluated in a clinical training set of enriched sera from 25 MPM, 25 HD and 25 NSCLC (Additional file 8: Table S5 and Additional file 9: Table S6) (3). Candidate biomarkers detected at higher abundance in sera of MPM were combined in logistic regression models to derive a multiplexed panel of six glycopeptides with optimal accuracy in discriminating MPM from HD. The panel was further confirmed in an independent validation set of 30 MPM, 29 HD and 28 NSCLC (Additional file 8: Table S5) together with the SRM-based monitoring of the biomarker mesothelin (Additional file 11: Table S7).

In our study, to identify new candidate biomarkers for MPM we performed quantitative discovery-driven screening of the surfaceome of MPM model cell lines. This has previously been proposed as a valuable source of MPM candidate biomarkers [18]. Subsequently, we used SRM assay technology to clinically evaluate the surfaceome-derived MPM candidate biomarkers in serum samples of suitably collected cohorts of MPM subjects and controls (Figure 1A and B).

## Results

### Quantitative analysis of mesothelin in serum

**Figure 2:**
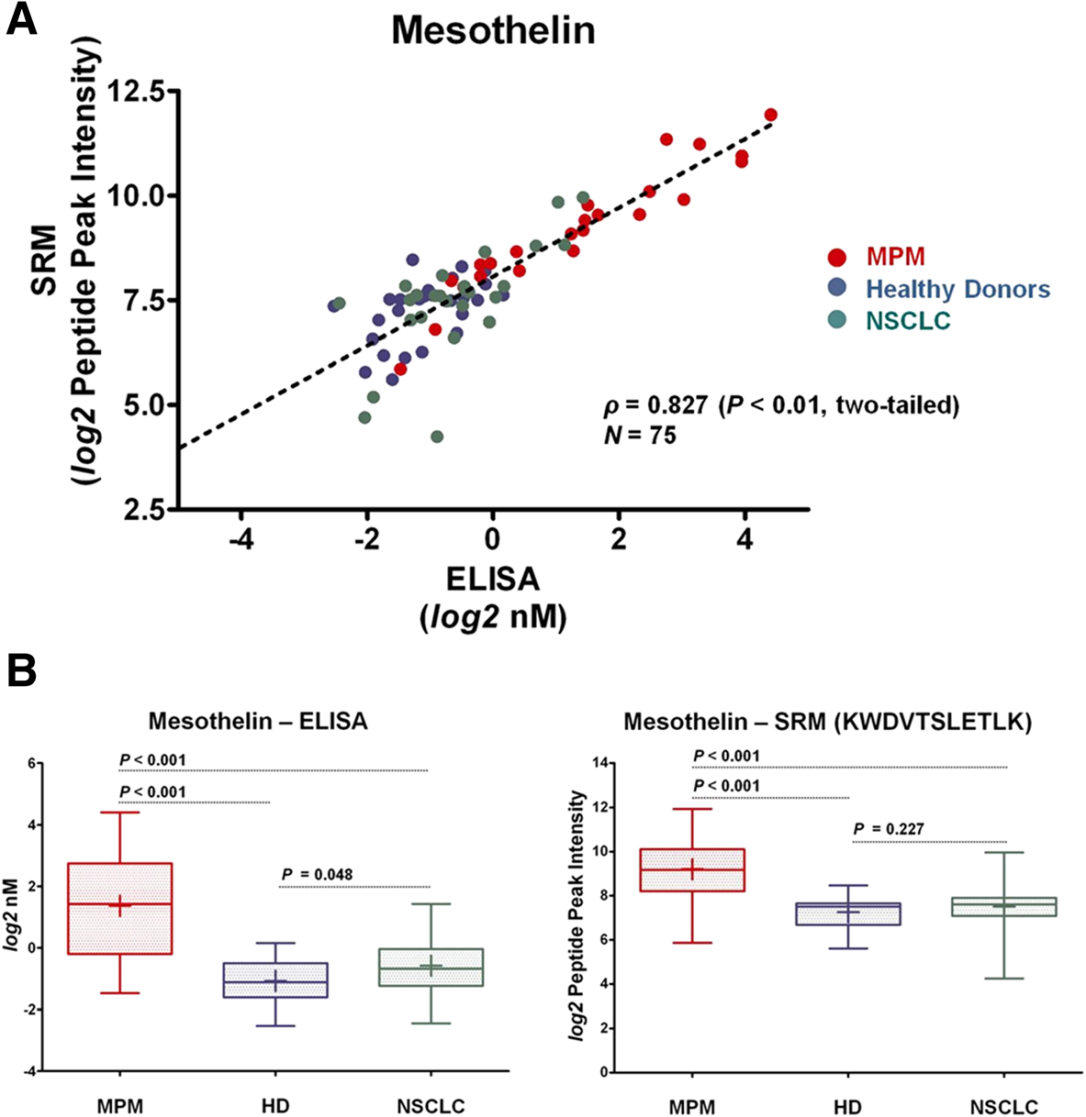
**Quantification of serum mesothelin. (A)** Correlation between the quantification of serum mesothelin by SRM assay technology (peptide KWDVTSLETLK) or by ELISA (Mesomark®) among 75 subjects (23 MPM = red dots, 26 HD = blue dots, 26 NSCLC = green dots) (Additional file 3: Table S2). Reported is the Spearman’s rank correlation coefficient (*P* < 0.05 is considered significant). Dashed line denotes best fit as calculated by linear regression (y = 8.1 + 0.82x). **(B)** Serum levels of mesothelin assessed by ELISA (Mesomark®) or by SRM assay technology (peptide KWDVTSLETLK) in the groups of MPM, HD and NSCLC of the 75 subjects (23 MPM, 26 HD, 26 NSCLC) (Additional file 3: Table S2). Significant differences of mean levels between the groups are assessed using the Mann-Whithney test. *P*-values are two-tailed and considered significant if < 0.05. Boxes report 25th and 75th percentile. Crosses inside the boxes indicate means and lines indicate medians. Whiskers indicate minimal and maximal values.

To verify the SRM based approach for MPM biomarkers in serum, we performed quantitative investigations of mesothelin in samples enriched for N-linked glycopeptides (N-glycopeptides). This enrichment step was chosen in our study to efficiently and reproducibly reduce the analytical complexity of the serum proteome and to focus our investigation on a sub-proteome particularly relevant for biomarker research, like represented by the N-linked glycoproteins (N-glycoproteins) [19-23]. To generate and optimize SRM-assays for the quantitative analysis of mesothelin, we followed the strategy proposed by Picotti et al. [24] using spectral libraries from chemically synthesized peptide-sequences. We established SRM-assays for the N-glycopeptides of membrane-bound mesothelin [25] (Additional file 1: Table S1) which is shed in serum [26,27] and for which a commercial ELISA-kit approved by the US Food and Drug Administration (FDA) (Mesomark®) [28,29] is available. We selected quantitative SRM-assays based on the N-glycopeptide KWDVTSLETLK (UniProt/Swiss-Prot: Q13421; D replaces the formerly glycosylated N after deamidation with the enzyme PNGaseF in the protocol for N-glycopeptide enrichment in serum [21]) which was routinely observed in our experiments (Additional file 2: Figure S1). We then quantified mesothelin using SRM assay technology in enriched serum samples from 75 subjects (23 MPM, 26 healthy donors, HD, and 26 non-small cell lung cancer, NSCLC, Additional file 3: Table S2). In parallel, in the same subjects we performed mesothelin quantification based on the FDA approved ELISA (Additional file 3: Table S2). The results showed firstly, a significant correlation between the quantification of mesothelin assessed by SRM at the glycopeptide level and by ELISA at the protein level (Figure 2A). Furthermore, differences in mean concentrations of mesothelin measured by ELISA among the groups of MPM, HD and NSCLC were reflected by SRM assay technology (Figure 2B). These results provided evidence for the ability of the SRM based approach in serum to accurately identify and quantify MPM biomarkers of clinical relevance.

### Surfaceome derived serum candidate biomarkers for malignant pleural mesothelioma

To identify candidate biomarkers for MPM, we performed a quantitative discovery-driven surfaceome screening in cell lines. To do so, we applied the mass spectrometry (MS) based Cell Surface Capture (CSC) technology [30] in two epithelioid and two biphasic MPM cell lines in parallel with two NSCLC (lung adenocarcinoma) and two non-cancerous pleural cell lines. A total of 668 N-glycopeptides were confidently (PeptideProphet probability ≥ 0.9) detected from more than 350 N-glycoproteins, which could potentially be shed into the blood stream. 514 N-glycopeptides were from MPM and 557 from non-MPM cell lines with 403 N-glycopeptides in common between the two groups. We prioritized candidate biomarkers potentially specific for MPM by focusing on N-glycopeptides reproducibly detected in higher abundance or in strong association with MPM cell lines. This screen led to the selection of 125 N-glycopeptides candidate biomarkers for MPM (Additional file 4: Table S3).

**Figure 3:**
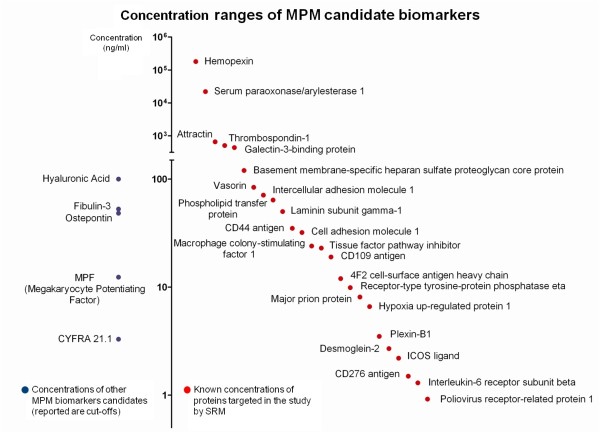
**Concentration ranges of proteins originating MPM candidate biomarker peptides detected through SRM-assays in serum.** Reported are protein concentrations in serum/plasma available from the PeptideAtlas database [32] (red dots) (Additional file 7: Table S4). For comparison, cut-off levels of selected MPM biomarkers in serum/plasma proposed by the literature [11,33-36] are reported (blue dots). Concentrations are reported in logarithmic scale.

Subsequently, we applied SRM assay technology for the multiplexed assessment of these candidate biomarkers in serum. To generate SRM-assays for the candidate biomarkers, we used again spectral libraries from chemically synthesized peptide-sequences and acquired spectra without confounding background matrices. The strategy enabled us to avoid potential MS platform related transition-selection biases [31] and sufficient peptide amounts for establishing optimized SRM-assays [24]. We obtained fragment spectra suitable for the library from 112 out of the initial 125 MPM candidate biomarkers and for these peptides we established initial SRM-assays in the background matrix of serum samples enriched for N-glycopeptides. We then performed multiplexed verification of the in serum unknown detectability of these candidate biomarkers by analyzing enriched serum samples of five MPM subjects (Additional file 5: Skyline file). In this initial screen we detected a total of 51 N-glycopeptides belonging to 36 N-glycoproteins which in the literature are reported at concentrations in serum/plasma between 180 μg/ml (hemopexin, UniProt/Swiss-Prot: P02790, peptide SWPAVGNCSSALR) and 0.92 ng/ml (poliovirus receptor-related protein 1, UniProt/Swiss-Prot: Q15223, peptide NPNGTVTVISR) (Additional file 6: Figure S2 and Additional file 7: Table S4) [32]. The majority of these proteins are reported at concentrations below 100 ng/ml and thus in the range of MPM biomarkers previously proposed in the literature [11,33,34] (Figure 3). Taken together, these observations supported the suitability and sensitivity of our targeted SRM-based approach for MPM biomarker investigation in serum.

### Seven glycopeptide signature for malignant pleural mesothelioma

**Figure 4:**
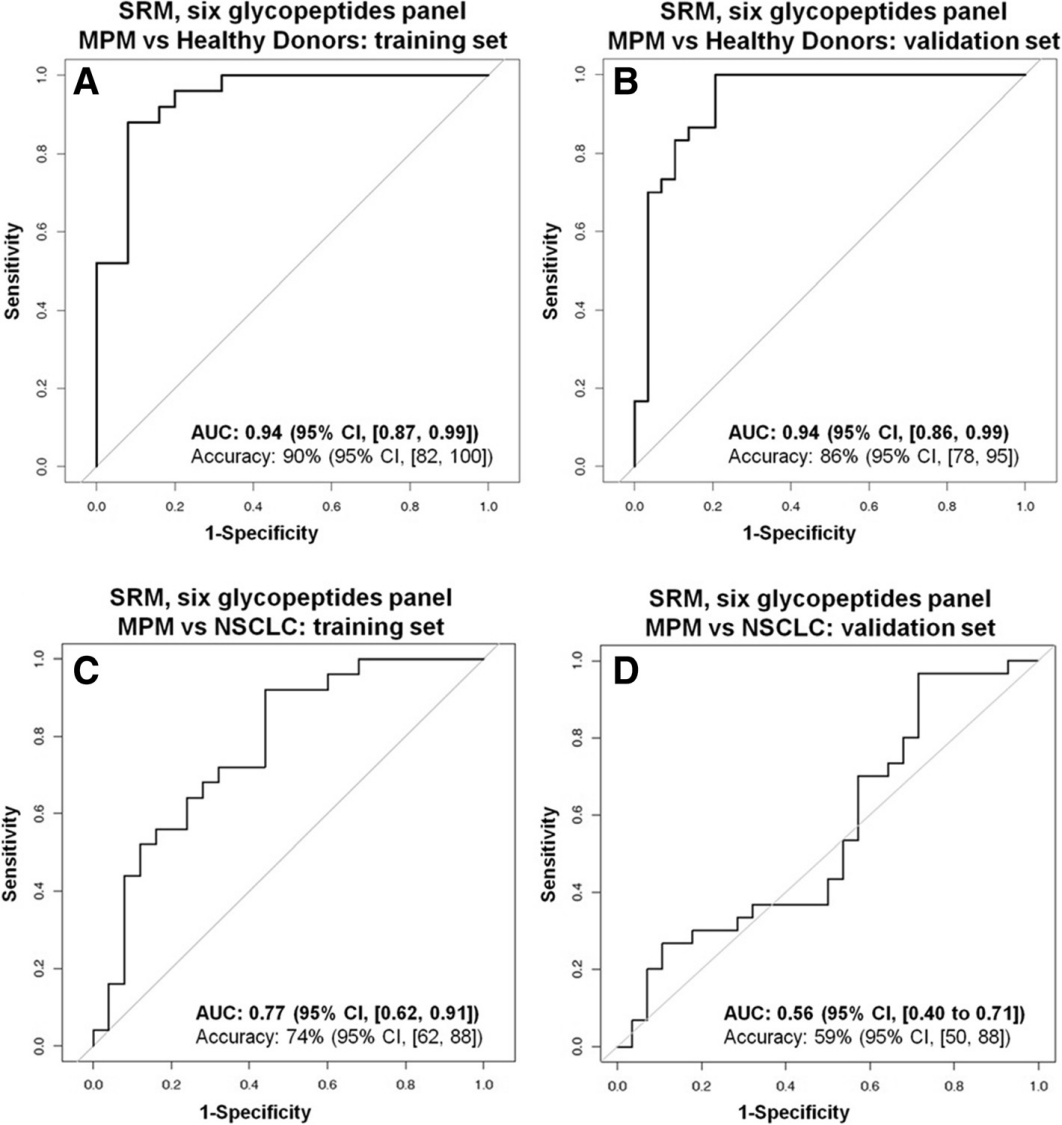
**ROC curves of the six glycopeptides panel.** Discrimination between MPM and HD **(A, B)** and MPM and NSCLC **(C, D)** in training **(A, C)** and validation **(B, D)** sets. Accuracies for MPM vs HD are at cut-off 0.61 and for MPM vs NSCLC at 0.35.

To assess the diagnostic potential of the 51 MPM candidate biomarkers, we relatively quantified them via optimized SRM-assays in an initial set of enriched serum samples from clinical cohorts of MPM (N = 25), HD (N = 25) and NSCLC subjects (N = 25) (clinical characteristics are reported in Additional file 8: Table S5). We used these samples as the training set for a predictive analysis. SRM assay technology allowed us to quantify all 51 MPM candidate biomarkers in parallel in one single injection per sample (Additional file 9: Table S6). Statistical significance analysis [37] identified four N-glycopeptides significantly higher in abundance in MPM as compared to HD and six N-glycopeptides of higher abundance in MPM as compared to NSCLC (Additional file 7: Table S4). We selected these ten peptides as candidate predictors in two separate logistic regression models for binary responses between MPM and HD or MPM and NSCLC. We than performed a stepwise selection of predictors and evaluation of their predictive ability using receiver operating characteristic (ROC) curves to identify a multiplexed panel of MPM candidate biomarkers with best discriminatory performance for MPM. The panel was composed by the six glycopeptides derived from the N-glycoproteins intercellular adhesion molecule 1 (UniProt/Swiss-Prot: P05362, peptide sequence ADLTVVLLR), basement membrane-specific heparan sulfate proteoglycan core protein (P98160, ALVDFTR), anthrax toxin receptor 1 (Q9H6X2, DFDETQLAR), serum paraoxonase/arylesterase 1 (P27169, HADWTLTPLK), hypoxia up-regulated protein 1 (Q9Y4L1, VIDETWAWK) and thrombospondin-1 (P07996, VVDSTTGPGEHLR). In the training set, the panel discriminated MPM from HD with an area under the curve (AUC) of 0.94 (95% confidence interval, CI, [0.87, 0.99]) and the highest accuracy (highest true positives and true negatives) of 90% (95% CI, [82, 100]) at a cut-off of 0.61 (Figure 4A). Subsequently, we confirmed the performance of the six glycopeptide panel in an independent validation set of 87 sera newly enriched for N-glycopeptides from MPM (N = 30), HD (N = 29) and NSCLC (N = 28) subjects (Additional file 8: Table S5). Here the panel discriminated MPM from HD with AUC of 0.94 (95% CI, [0.86, 0.99]) and accuracy of 86% (95% CI, [78, 95]) at the 0.61 cut-off (Figure 4B). However, the panel failed to discriminate MPM from NSCLC: AUC in the training set was 0.77 (95% CI, [0.62, 0.91]) with accuracy of 74% (cut-off 0.35; 95% CI, [62, 88]) (Figure 4C) and in the validation set was 0.56 (95% CI, [0.40, 0.71]) with accuracy of 59% (cut-off 0.35; 95% CI, [50, 88]) (Figure 4D).

We compared the discriminatory performance of the six glycopeptides panel to that of the FDA approved ELISA assay for mesothelin (Mesomark®) [28] (Additional file 10: Figure S3). In the 75 subjects above (23 MPM, 26 HD and 26 NSCLC, Additional file 3: Table S2) for which ELISA measurements were available, mesothelin ELISA discriminated MPM from HD with AUC of 0.92 (95% CI, [0.83, 0.99]) and accuracy of 82% (95% CI, [71, 94]) at the 2 nM cut-off proposed in the literature [10]. In the same subjects, which were part of the validation set above, the six glycopeptides panel based on SRM assay technology had AUC of 0.94 (95% CI, [0.86, 0.99]) and accuracy of 86% (95% CI, [71, 96]) at the 0.61 cut-off, indicating a discriminatory power similar to that of the ELISA assay. For the discrimination between MPM and NSCLC, mesothelin ELISA was superior to SRM and had AUC of 0.84 (95% CI, [0.71, 0.94]) whereas the six glycopeptides panel had AUC of 0.54 (95% CI, [0.37, 0.71]).

**Figure 5:**
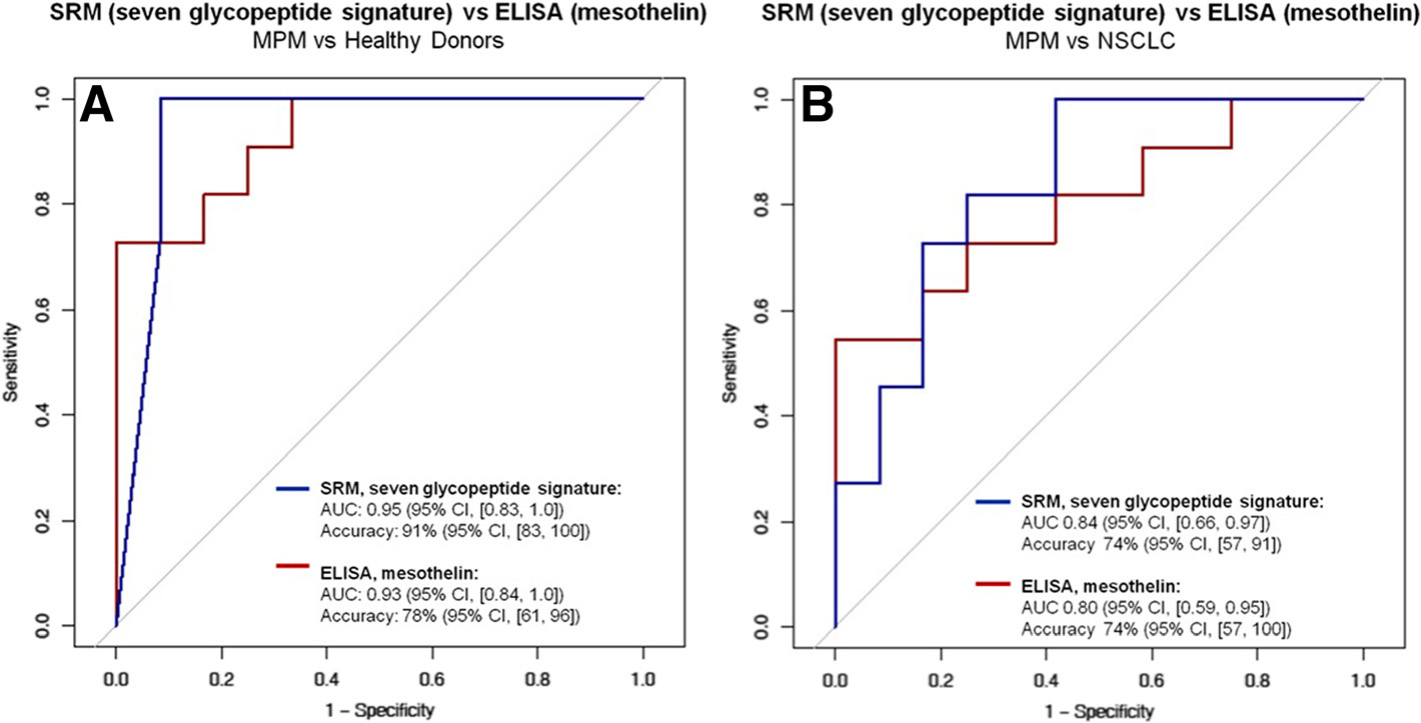
**ROC curves of the seven glycopeptide MPM signature.** Discrimination between MPM and HD **(A)** and MPM and NSCLC **(B)** using the SRM-based seven glycopeptide signature or using mesothelin ELISA. Parameterization of the seven glycopeptide signature was performed in a sub-training set (12 MPM, 14 HD and 14 NSCLC) and applied to a sub-validation set (11 MPM, 12 HD and 12 NSCLC) of the group of 75 subjects for which mesothelin ELISA (Mesomark®) measurements were available. Reported are AUCs for the seven glycopeptide signature (blue line) and mesothelin ELISA (red line) in the sub-validation set. Accuracy of the seven glycopeptide signature is at cut-off 0.5 for MPM vs HD and 0.6 for MPM vs NSCLC. Accuracy for mesothelin ELISA is at 2 nM cut-off. CI indicates a 95% confidence interval.

From this last observation, we argued that the specificity of the SRM panel for MPM could be increased by including mesothelin. Thus, we added the SRM monitoring of mesothelin peptide KWDVTSLETLK to the signature (Table 1 and Additional file 11: Table S7) and tested it in the group of 75 subjects for which mesothelin ELISA measurements were available for comparison. We performed parameterization of the signature using a sub-training set of 12 MPM, 14 HD and 14 NSCLC and assessed its performance in a sub-validation set of 11 MPM, 12 HD and 12 NSCLC (Figures 5A and B). Here, the SRM based signature discriminated MPM from HD with AUC of 0.95 (95% CI, [0.83, 1.0]) and highest accuracy of 91% (95% CI, [83, 100]) and MPM from NSCLC with AUC of 0.84 (95%CI, [0.66, 0.97]) and highest accuracy of 74% (95% CI, [57, 91]). The performance of the signature was higher than for mesothelin ELISA, which in the same subjects had AUC of 0.93 (95% CI, [0.84, 1.0]) and accuracy of 78% (95% CI, [61, 96]; cut-off 2 nM) in discriminating MPM from HD and AUC of 0.80 (95% CI, [0.59, 0.95]) and accuracy of 74% (95% CI, [57, 100]; cut-off 2 nM) in discriminating MPM from NSCLC.

**Table 1:** Seven glycopeptide signature for MPM in serum

MPM candidate biomarker N-glycopeptides monitored by SRM*	**Protein name**	**UniProt/Swiss-Prot entry**	**Gene name**	ng/ml**
**ADLTVVLLR**	Intercellular adhesion molecule 1	P05362	ICAM1	71
**ALVDFTR**	Basement membrane-specific heparan sulfate proteoglycan core protein	P98160	HSPG2	120
**DFDETQLAR**	Anthrax toxin receptor 1	Q9H6X2	ANTXR1	Not available
**HADWTLTPLK**	Serum paraoxonase/arylesterase 1	P27169	PON1	22*'*000
**VIDETWAWK**	Hypoxia up-regulated protein 1	Q9Y4L1	HYOU1	6.6
**VVDSTTGPGEHLR**	Thrombospondin-1	P07996	THBS1	510
**KWDVTSLETLK**	Mesothelin	Q13446	MSLN	Not available

*D replaces the former glycosylated N in the natural peptide sequence after deamidation induced by PNGaseF treatment.

**Concentration in serum/plasma as estimated in PeptideAtlas (ref. Farrah et al. [32]).

## Discussion

The development of protein biomarkers in serum requires the availability of reliable analytical tools for the unbiased prioritization and large scale clinical testing of novel candidates [38,39]. ELISA’s assays for biomarker investigation are typically obtainable only for a subset of candidates and establishing new ELISA’s solely for the purpose of testing candidates is time consuming and too expensive [40,41]. In our study, we presented the application of targeted proteomics SRM assay technology in serum for the investigation and clinical evaluation of candidate biomarker panels for MPM. The approach presented itself as an accurate alternative to immunoassays and allowed us to follow a hypothesis driven biomarker investigation independently of the still piecemeal availability of antibodies.

The underlying hypothesis of our investigation was that the surfaceome of MPM can reveal novel blood accessible biomarkers. This was suggested by the fact that the cell surface proteins are exposed to the tumor environment and thus prone to be shed or released to the stroma and finally collected into the blood [42,43]. Indeed, many proposed blood tumor-markers like mesothelin, carcinoembryonic antigen (CEA) or cancer antigen 125 (CA-125) are glycoproteins of cell surface origin. Following this hypothesis, our SRM investigation in serum detected 51 out of the 112 surfaceome derived candidate biomarkers. This was a considerable fraction of the candidates, considering that they were selected without prior knowledge and from extremely simplified tumor models as represented by cell lines. Interestingly, the majority of the candidates were at concentrations in the range of proposed MPM biomarkers like fibulin-3, megakaryocyte potentiating factor (MPF) or osteopontin. This observation, together with the SRM detection of mesothelin, confirmed that our targeted proteomics strategy could reliably access that fraction of the serum proteome which seems to be of particular relevance for MPM biomarker investigations.

In our study, if available, at least two surfaceome detected N-glycopeptides per protein were initially investigated by SRM in serum. For our final MPM signature in serum we selected the best peptide for a particular protein, e.g. these peptides were most consistently detected. Potential variations in the detectability and response factor of peptides from the same proteins are related to a number of reasons. One reason is certainly related to the fact that each peptide has its peculiar physicochemical characteristics which will influence its mass spectrometric detection independently of a common protein of origin [44,45]. At the same time it is also likely that the signature reflects the complexity of a natural tissue environment [46,47]. Indeed, several biochemical and proteolytic processes are expected to take place in the tumor microenvironment which can modify the original structure of the cell surface proteins [48,49]. It is thus likely that not intact proteins but rather only fragments of them will reach and pass the vessel barriers [50,51]. This could at least in part explain the apparently asynchronous behavior in serum of peptides from the same protein.

Despite the confident discrimination between MPM and healthy, our study cannot conclusively answer the question if the candidate biomarkers of the signature are MPM specific or rather more generally cancer associated. Indeed, without mesothelin, the six biomarkers of the signature failed to discriminate MPM from NSCLC and their association with other tumors is reported [52-56]. Nevertheless, the SRM signature inclusive of mesothelin presented accuracies higher than the ELISA test for the single marker mesothelin. This indicated that the integration of the seven MPM biomarkers in the multiplexed SRM signature could complement the limited sensitivity of mesothelin, taking at the same time advantage of its specificity for MPM. Here, we have to point out that, because of the exploratory nature of our investigation, the majority of the patients included in our study were at advanced disease stages and that controls did not include confounding conditions like chronic inflammations or other non-malignant pathologies of the lung. As a consequence, the accuracy of the MPM signature could be lower if applied to more heterogeneous populations, like indirectly suggested by the higher AUCs of mesothelin ELISA of our study in respect to literature reports [10].

Finally, it has to be highlighted that the MPM signature includes hypoxia up-regulated protein 1 (also known as ORP150 or GRP170; UniProt/Swiss-Prot: Q9Y4L1, gene name HYOU1) which is a heat shock protein with chaperone function in the endoplasmic reticulum [57]. This could arise some concern about the specificity of our approach. It is therefore worthwhile to mention here that, in accord with other groups [58,59], in our surfaceome experiments we reproducibly observed the protein and it is known that heat shock proteins can be expressed on cell surfaces or be secreted to blood [60-63].

## Conclusions

In conclusion, the SRM assay technology based approach chosen for our clinical MPM investigation allowed us to directly evaluate a larger set of candidate serum biomarkers resulting in a seven glycopeptide signature with diagnostic potential for MPM. Our results indicate that the SRM assay technology lends itself for the fast clinical evaluation of candidate biomarkers in serum. In this respect, larger SRM-assays repositories are currently being generated [64,65], which will ultimately enable the quantitative evaluation of biomarker candidates of interest in the disease setting of choice.

## Methods

### Cell culture

The MPM cell lines ZL55, SDM4, SDM5 and SDM34 were from surgical tumor samples and the pleural cell line SDM104 was from a surgical biopsy of a patient with chronic pleuritis. Cell lines were established as previously described [66,67] and were from patients with pathologically confirmed diagnosis and treated at the University Hospital Zürich. HCC4012 was from human mesothelial cells immortalized with hTERT (kind gift of Dr. A. Gazdar, The University of Texas, Southwestern Medical Center). ADCA cell lines Calu-3 and SK-LU-1 were from American Type Culture Collection (ATCC; Manassas, VA). Detailed growing conditions can be found in Additional file 12: Supplementary Methods.

### CSC-based surfaceome analysis and MPM candidate biomarkers selection

CSC followed by MS analysis was performed as described previously [30]. For label free relative-quantification, raw data of duplicate measurements were acquired in profile mode on a Fourier-Transform LTQ MS (FT-LTQ; Thermo Electron, San Jose, CA), converted to mzXML [68] and analyzed with the software Superhirn [69]. For sequence identification MS/MS spectra of centroided raw files were converted to mzXML and searched against the IPI Human database v3.26 using the search algorithm SEQUEST v27 [70]. Criteria for MPM candidate biomarker peptides were: *1.* fully tryptic. *2.* deamidation of asparagine in the consensus sequence NxS/T (x denotes any amino acid excluded proline) after treatment with PNGaseF. *3.* PeptideProphet probability ≥ 0.9. *4.* sequence proteotypic and unique for proteins reviewed in Uniprot [71] and with subcellular localization associated to membranes or secreted. *5.* reproducibly higher abundant in MPM in at least two MPM vs non-MPM cell lines comparisons, or originating from the same protein of an higher abundant peptide, or deriving from a protein not observed in non-MPM cell lines but detected in MPM at least in two cell lines or with two peptides. Further details about quantitative CSC analysis are reported in Additional file 12: Supplementary Methods.

### Generation of SRM-assays

To establish glycopeptide-specific SRM assays, spectra of MPM candidate biomarker glycopeptides were generated by using synthetic heavy isotope-labeled (heavy, with R ^13^C_6_/^15^ N_4_ and/or K ^13^C_6_/^15^ N_2_) peptides (SpikeTides_L™, JPT Peptide Technologies, Berlin, Germany) with aspartic acid (D) replacing the putative glycosylated asparagines (N) according to the mass modification introduced by treatment with the enzyme PNGaseF in the protocol for enrichment of N-glycopeptides from serum. Spectra were acquired on Quadrupole Time-of-Flight (QTOF) LC/MS series 6520 or 6550 instruments (Agilent Technologies, Santa Clara, CA) equipped with an HPLC-Chip Cube interface (Agilent Technologies) and operated in data dependent mode. MS/MS spectra were used to generate initial SRM-assays for MPM candidate biomarkers. They consisted of at least six transitions per peptide selected based on signal intensities of heavy peptides (SpikeTides_L™, JPT Peptide Technologies) spiked in the matrix of enriched serum. SRM-assays of candidate biomarkers detected in serum were further individually optimized and consisted of four transitions per peptide with at least three fixed transitions used for quantification. Details about spectra acquisition, MS settings, SRM-assays generation and optimization can be found in Additional file 12: Supplementary Methods. All assays developed can also be downloaded in form of a Skyline library file (Additional file 5: Skyline file).

### Serum samples

Whole blood samples were obtained after written informed consent from therapy naïve patients with pathologically proven diagnosis of MPM or NSCLC and treated at the University Hospital Zürich. Staging was based on TNM-International Union Against Cancer (UICC, sixth edition) selecting the highest stage in case of ambiguous report. Whole blood samples from HD were from blood donors at the Blood Transfusion Service Zürich, SRC, Schlieren, Switzerland and judged healthy based on standardized medical questionnaire [72]. The study was approved by the Ethics Committee of the University Hospital Zürich. Serum processing is reported in Additional file 12: Supplementary Methods.

### Serum enrichment for N-glycopeptides and MS analysis

For SRM analysis, 100 μl of serum were enriched for N-glycopeptides using a modified protocol of the method for solid phase extraction of N-glycopeptides (SPEG) [21]. 1.5 μl of peptide mixture were analyzed on a QQQ LC/MS 6460 series (Agilent Technologies) equipped with an HPLC-Chip Cube interface (Agilent technologies) and using a nano-flow gradient of 5 to 35% acetonitrile (ACN) /water, 0.1% formic acid (FA) over 30 min. The software Skyline [73] was used for SRM-traces visualization after Savitzky-Golay smoothing, SRM-methods building and calculation of peak transition-intensities. Details about serum processing and MS settings can be found in Additional file 12: Supplementary Methods.

### Verification of MPM candidate biomarker peptides in serum

To verify the detectability of MPM candidate biomarker peptides in serum, samples from five MPM subjects were enriched for N-glycopeptides and analyzed on a QQQ LC/MS instrument using not-optimized SRM-assays. Sample processing and MS settings were as described above. Transitions were monitored in scheduled SRM-mode allowing for a maximum of 339 total transitions and 176 concurrent transitions per method. Cycle-times ranged from 2 to 4.1 s allowing for a minimal dwell time of 18.5 ms per transition. Delta retention time window was 4 or 5 min. Confident detection of MPM candidate biomarker peptides in serum was manually confirmed based on transition co-elution with simultaneously monitored heavy isotope-labeled synthetic peptides with matching sequences (SpikeTides_L™, JPT Peptide Technologies) spiked in the samples before MS analysis.

### SRM analysis of candidate biomarker N-glycopeptides from clinical cohorts

Serum samples of training and validation sets were enriched for N-glycopeptides and analyzed using optimized SRM assays on a QQQ LC/MS instrument as described above. The two sets were processed and analyzed at separate time points. Samples of the same set were processed simultaneously in randomized order and analyzed in technical duplicates on the QQQ. Eleven samples (normalizing-samples) from the training set were re-processed and re-analyzed in parallel with the validation set and results were used for normalization of SRM signals between the two groups. These samples were subsequently excluded from the validation set. For relative quantification, a mix of heavy isotope-labeled synthetic peptides with sequences matching the MPM candidate biomarker peptides was used as internal standard (SpikeTides_L™, JPT Peptide Technologies, for mesothelin heavy isotope-labeled synthetic peptides were from Thermo Scientific) and spiked at fixed concentration in each sample before MS analysis at a volume ratio of 1:5 of heavy-peptide-mix to serum sample. To assess technical variations among runs, iRT peptides (Biognosys, Schlieren, Switzerland) [74] were spiked in each sample before MS analysis. MS analysis of serum samples from the training set was performed using a scheduled SRM method including a total of 468 light and heavy transitions. Cycle time was of 3.7 s allowing for the acquisition of at least eight data points per peptide elution profile. RT window was set to 5 min. Dwell time per transition ranged from a minimum of 16 ms to a maximum of 459 ms. The number of concurrent transitions ranged from 8 to maximal 190. Samples of the validation set were analyzed in scheduled SRM-mode monitoring for a total of 288 light and heavy transitions. Cycle time was set to 3 s for the acquisition of at least eight data points per peptide elution profile using a delta RT window of 5 min. Minimal dwell time per transition was 26.6 ms and maximal was 459 ms. Minimal and maximal number of concurrent transitions were 8 and 123 respectively. Both method included transitions from the iRT peptides and peptides of the serum proteins haptoglobin (UniProt entry P00738) and kininogen-1 (UniProt entry P01042) used as internal reference control for sample handling and MS performance. Confident detection of MPM candidate biomarker peptides was confirmed manually based on transition co-elution with heavy isotope-labeled internal standards.

### Statistical significance analysis and prediction analysis

Statistical analysis of peptide differential abundance utilized SRMstats package in R [37,75]. Ten peptides of higher abundance in training set in either comparison for MPM vs. HD or MPM vs. NSCLC were further used in two logistic regression models for MPM vs HD and MPM vs NSCLC. In order to account for relative experimental yield and reproducibility of sample preparation between training and validation sets, we developed a two-step normalization procedure based on the eleven normalizing-samples that were present in both sets. The first normalization step accounted for variations in the mass spectrometer performance, separately for the training and the validation sets, by equating median intensities of reference transitions between the runs. The second normalization step shifted the intensities of the endogenous transitions in the validation set to the scale of the training set. Specifically, for each endogenous transition we calculated the median difference of log-intensities among the eleven normalizing-samples in the validation and the training sets. The difference was then subtracted from the endogenous intensities in all the validation samples. All inputs for the logistic regressions are estimates of peptide abundance in each biological sample on a relative scale, which are summarized across multiple transitions and technical replicate runs. This summarization was performed in SRMstats fitting logistic regression in R. 'pROC’ package in R was used to draw ROC curves and to calculate AUCs and CI with bootstrap methods [76]. Correlations and Mann-Whithney test were calculated and visualized using IBM SPSS Statistics Standard v17.0 (SPSS, Inc, Chicago, IL) or GraphPad Prism 5 (GraphPad Software, Inc, San Diego, CA).

### ELISA

Mesothelin ELISA in serum was performed in duplicates using the Mesomark-kit™ (Fujirebio Diagnostic, Malvern, PA) according to the manufacturer’s protocol. Averaged values were used for analysis. Samples with coefficient of variation > 15% were excluded.

## Competing interests

The authors declare that they have no competing interests.

## Authors’ contributions

FC, RA, EFB, RS and BW conceived the project, designed and interpreted experiments. FC and AN performed experiments. DBF provided support and interpreted CSC experiments. MC and OV performed statistical analysis in clinical serum cohorts. FC, EFB, RS and AZ coordinated clinical sample acquisition, FC and AZ processed and registered samples. FC, MC and BW wrote the manuscript. All authors read and approved the final manuscript.

## Supplementary Material

Additional file 1: Table S1 Optimized SRM-assays for mesothelin N-glycopeptides monitored by SRM in serum. Click here for file

Additional file 2: Figure S1 SRM detection of mesothelin in serum. Click here for file

Additional file 3: Table S2 SRM and ELISA quantitative investigation of mesothelin in serum samples of 75 subjects. Click here for file

Additional file 4: Table S3 A. Surfaceome peptides fulfilling requirements for MPM candidate biomarker. B. MPM candidate biomarkers selected for SRM analysis in serum. Click here for file

Additional file 5 **Skyline file.** (Skyline, https://brendanx-uw1.gs.washington.edu/labkey/project/home/software/Skyline/begin.view). Skyline library file and serum verification of SRM-assays developed for surfaceome derived MPM candidate biomarkers. Click here for file

Additional file 6: Figure S2 MPM candidate biomarker peptides detected by SRM in the screening of five MPM sera after enrichment for N-glycopeptides. Click here for file

Additional file 7: Table S4 MPM candidate biomarker peptides identified by SRM in sera enriched for N-glycopeptides. Click here for file

Additional file 8: Table S5 Clinical characteristics of the 55 MPM, 53 NSCLC and 54 healthy donors in the training and validation set. Click here for file

Additional file 9: Table S6 Optimized SRM-assays for the MPM candidate biomarkers detected in serum. Click here for file

Additional file 10: Figure S3 Six glycopeptides panel vs mesothelin ELISA. Click here for file

Additional file 11: Table S7 Transitions of the seven glycopeptide signature for MPM. Click here for file

Additional file 12 Supplementary methods. Click here for file
