# Supplementary material for: Identification of a seven glycopeptide signature for malignant pleural mesothelioma in human serum by selected reaction monitoring
Source: Clin Proteomics. 2013 Nov 8;10(1):16. doi: 10.1186/1559-0275-10-16 (PMC3827840; doi:10.1186/1559-0275-10-16)
Supplement: Additional file 2: Figure S1 — SRM detection of mesothelin in serum. [file 1559-0275-10-16-S2.doc]

**Additional file 2 to *Cerciello et al.* : Figure S1**

**S1A**

**
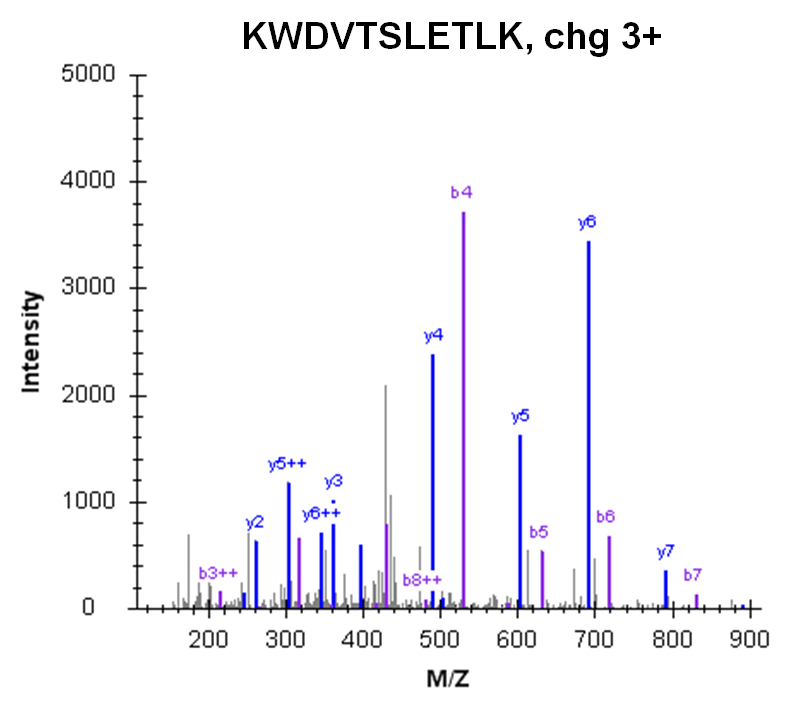
**

**S1B**

**
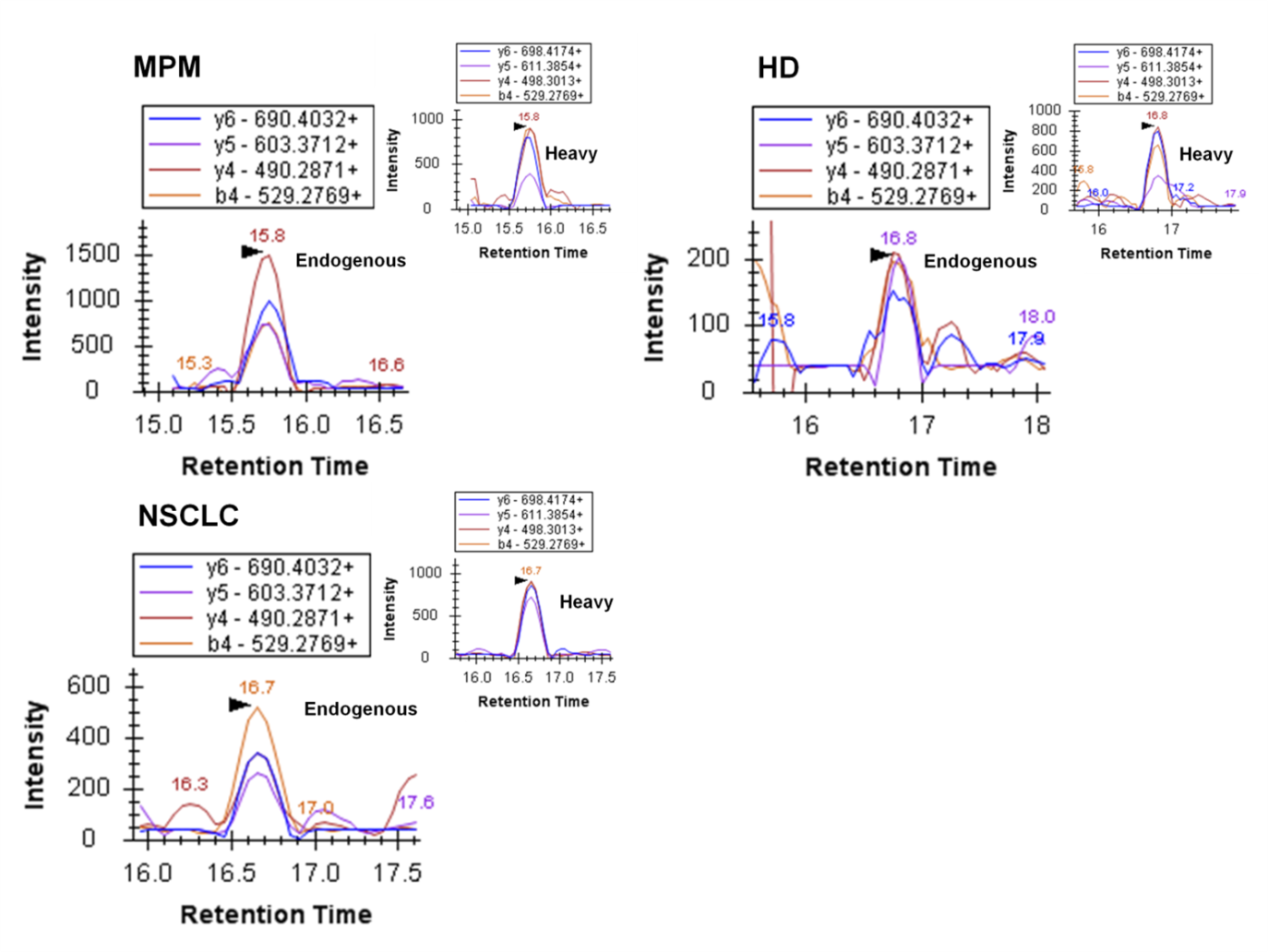
**

**S1C**

**
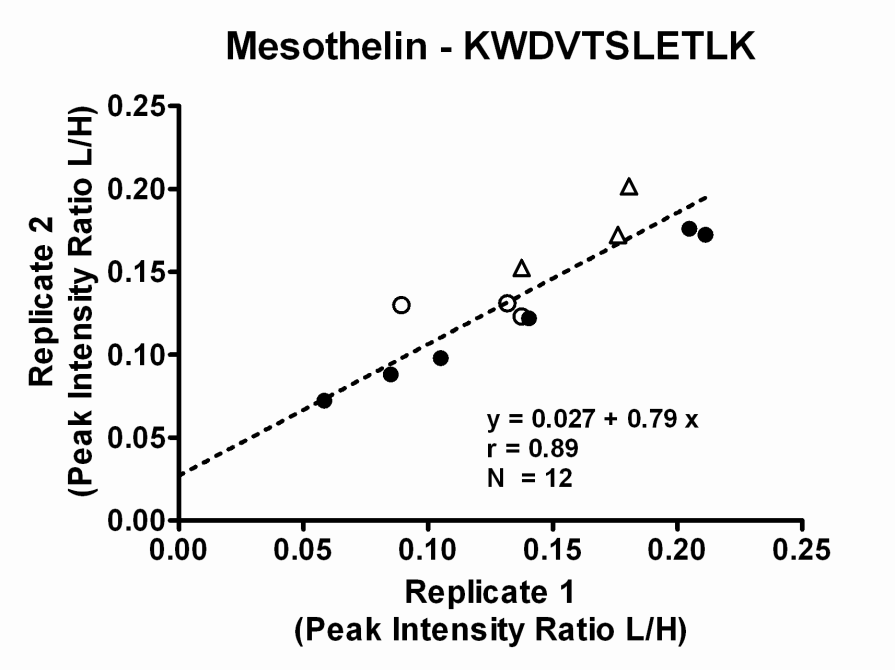
**

**Figure S1. SRM detection of mesothelin in serum.** (**S1A**) MS/MS spectrum of the synthetic mesothelin peptide KWDVTSLETLK used for generation and optimization of mesothelin SRM-assays. (**S1B**) SRM-assays traces of mesothelin peptide KWDVTSLETLK detected by SRM assay technology in serum of MPM, HD and NSCLC (label: Endogenous). Shown are also SRM-assays traces of the isotopic labeled internal control with matching sequence used for relative-quantification among samples (label: Heavy). (**S1C**) Replicate SRM analysis of the mesothelin peptide KWDVTSLETLK (charge state 3+) in serum. SRM measurements are performed in the sera of eight MPM subjects. In the case of six subjects, sera were enriched in duplicates for N-glycopeptides (black dots) and in the case of two subjects sera were enriched in triplicates at different time points (circles respectively triangles). Peak-signal-intensities of the transitions are summed and reported is the light-to-heavy (L/H) ratio between the endogenous and the heavy peptide spiked-in as internal control. Reported is the mean of three repeated measurements per replicate. Pearson correlation coefficient was *r* = 0.89 (95% CI, [0.65, 0.97], *p* < 0.0001, two tailed) and *r* squared was 0.79. Dashed line denotes the line of best fit calculated by Deming regression analysis and the equation is reported. Slope was 0.79 ± 0.13 (95% CI*,* [0.51, 1.1]), intercept 0.027 ± 0.019 (95% CI, [-0.014, 0.068]).
