# Supplementary material for: Identification of a seven glycopeptide signature for malignant pleural mesothelioma in human serum by selected reaction monitoring
Source: Clin Proteomics. 2013 Nov 8;10(1):16. doi: 10.1186/1559-0275-10-16 (PMC3827840; doi:10.1186/1559-0275-10-16)

Additional file 6 to *Cerciello et al.* : Figure S2

Figure S2 (following pages)**. MPM candidate biomarker peptides detected by SRM in the screening of five MPM sera after enrichment for N-glycopeptides.** Shown are the MS/MS spectra (y+ , y++ and b+, b++ ions) of synthetic heavy isotope-labeled peptides used for the generation of SRM-assays (panel left). Shown are also SRM-traces (three to four best transitions) of the endogenous peptide detected in serum (lower panel right, labeled: Endogenous) together with their matching heavy isotopic-labeled internal standard (upper panel right, labeled: Heavy). Notice that in the peptide sequences monitored by SRM, D (aspartic acid) replaces the naturally occurring glycosylated N (asparagine) because of the deamidation of the asparagine residues after treatment with the enzyme PNGaseF. (**S2A**) MPM candidate biomarker peptides confidently detected in serum. (**S2B**) MPM candidate biomarker peptides with difficult detection in serum.

S2A

Heavy

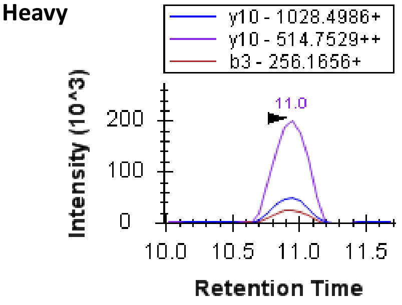

Galectin-3-binding protein

UniProt Entry: Q08380
Gene Name: LGALS3BP

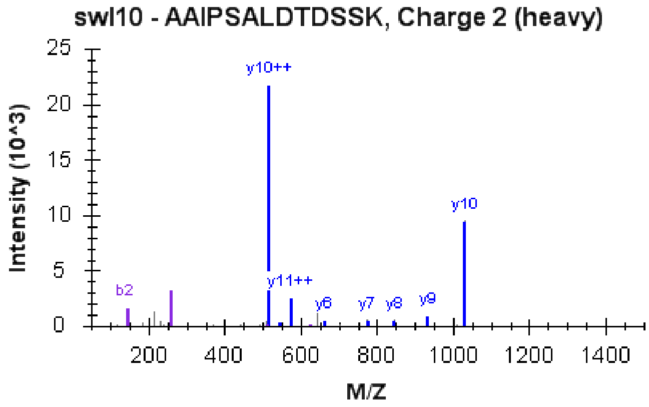

Endogenous

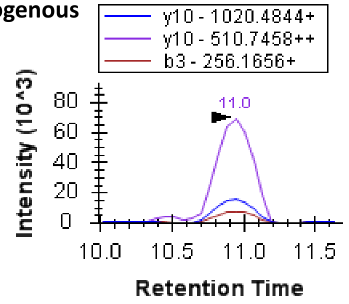

Heavy

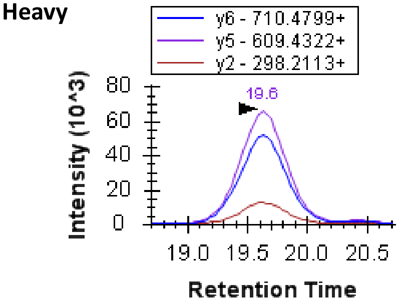

Intercellular adhesion molecule 1

UniProt Entry: P05362
Gene Name: ICAM1

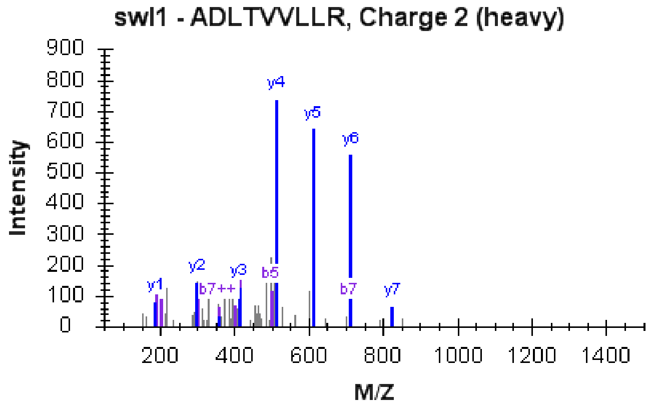

Endogenous

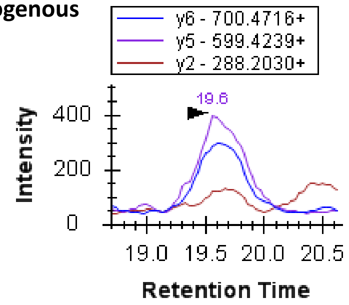

13

S2A

CD44 antigen

UniProt Entry: P16070
Gene Name: CD44

swl10 - AFDSTLPTMAQMEK, Charge 2 (heavy)

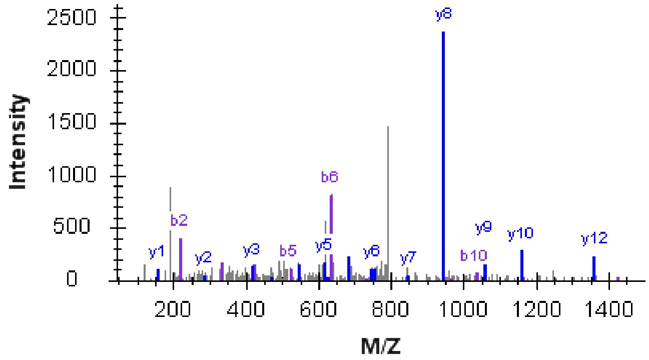

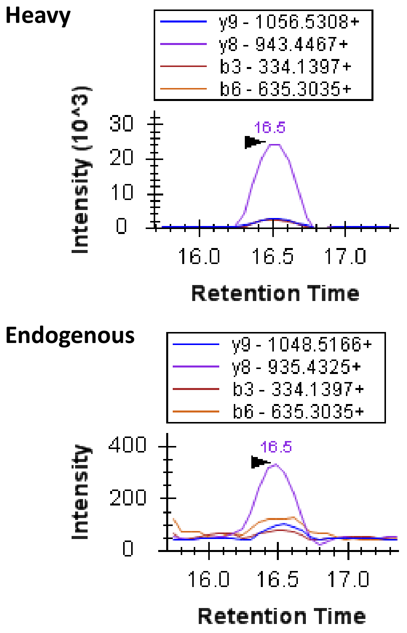

Galectin-3-binding protein

UniProt Entry: Q08380
Gene Name: LGALS3BP

swl10 - ALGFEDATQALGR, Charge 2 (heavy)

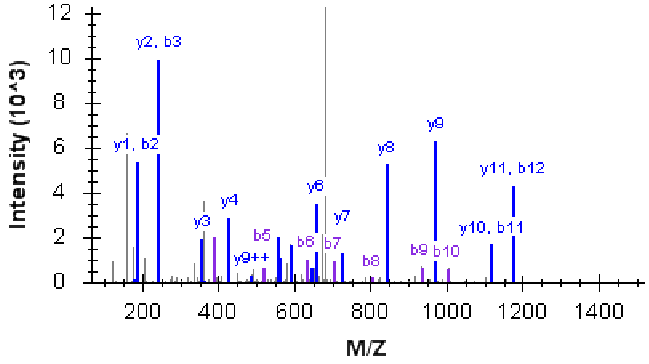

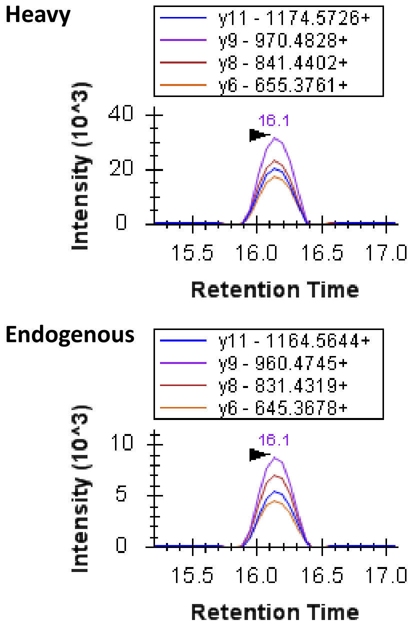

S2A

Basement membrane-specific heparan sulfate proteoglycan core protein

UniProt Entry: P98160
Gene Name: HSPG2

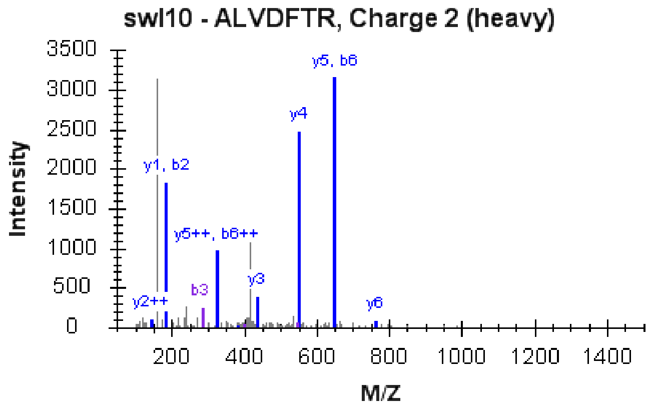

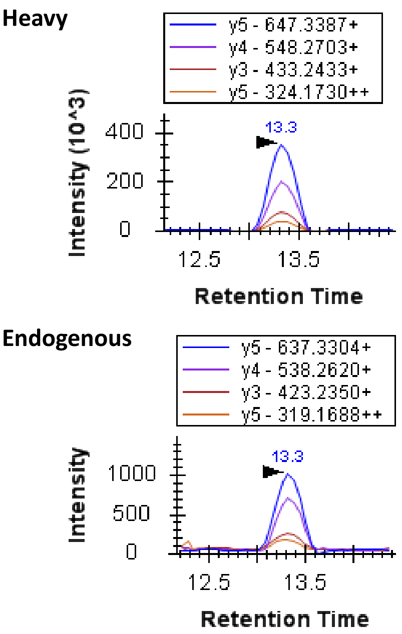

Galectin-3-binding protein

UniProt Entry: Q08380
Gene Name: LGALS3BP

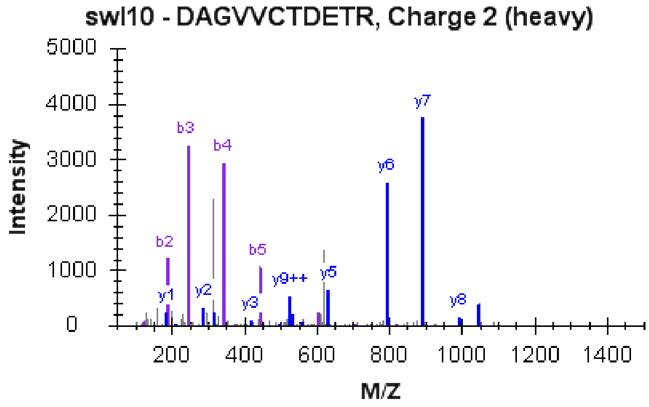

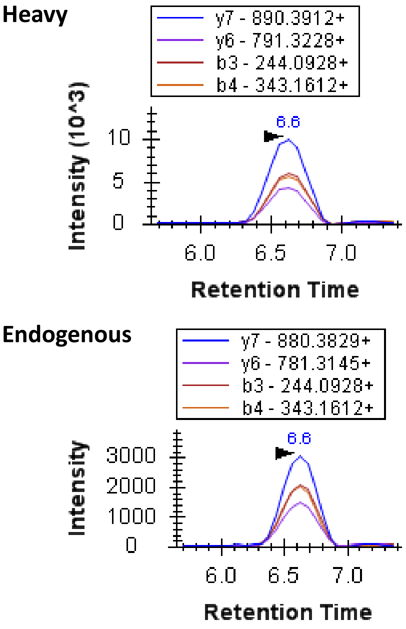

S2A

4F2 cell-surface antigen heavy chain

UniProt Entry: P08195
Gene Name: SLC3A2

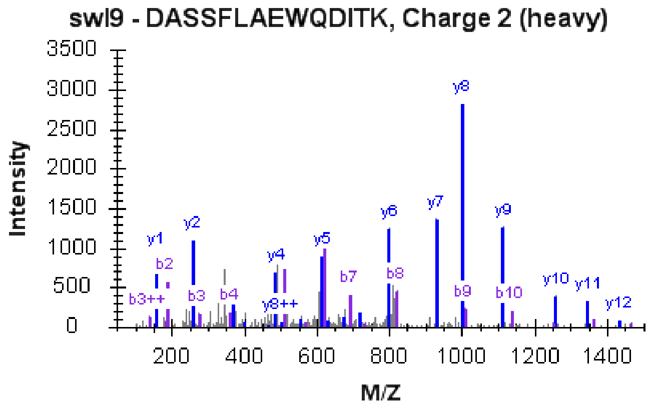

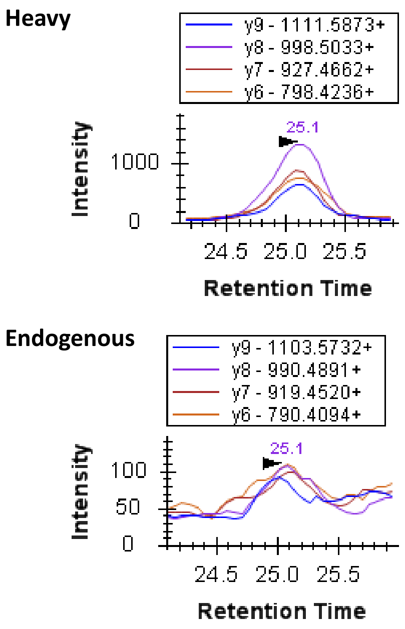

Anthrax toxin receptor 1

UniProt Entry: Q9H6X2
Gene Name: ANTXR1

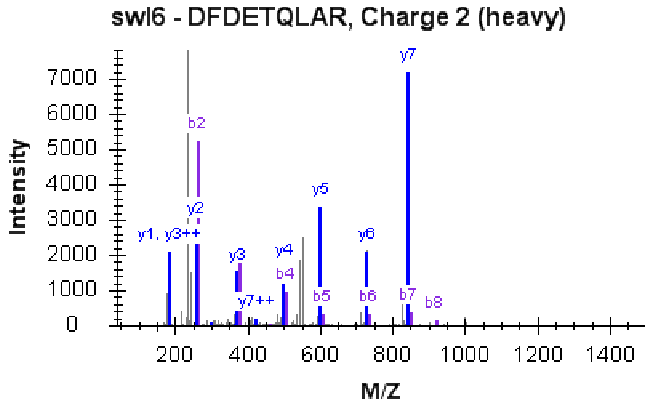

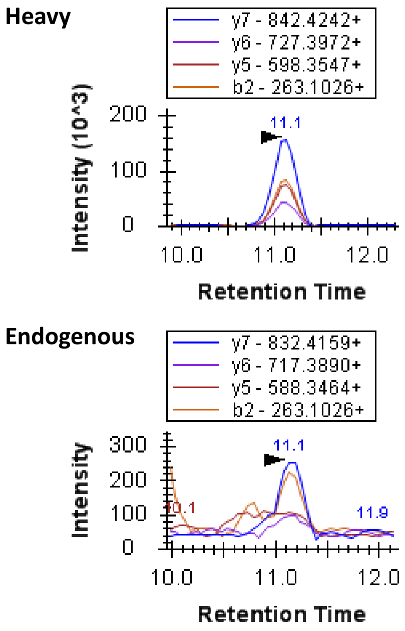

S2A

Teneurin-2

UniProt Entry: Q9NT68
Gene Name: ODZ2

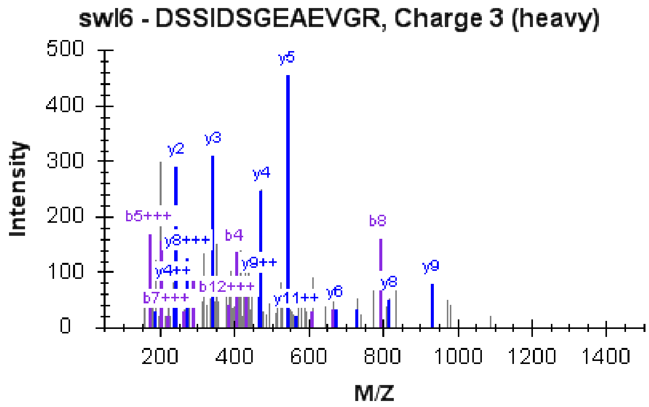

Heavy

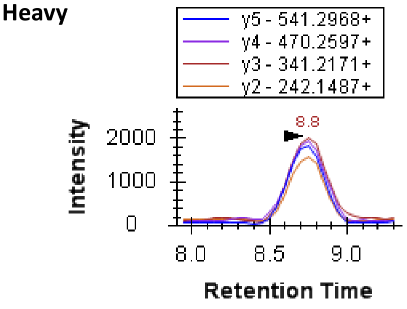

Endogenous

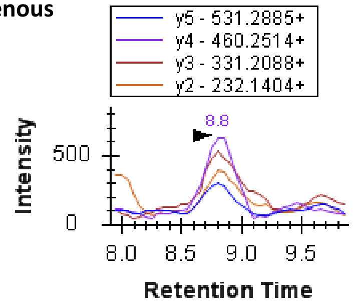

Desmoglein-2

UniProt Entry: Q14126
Gene Name: DSG2

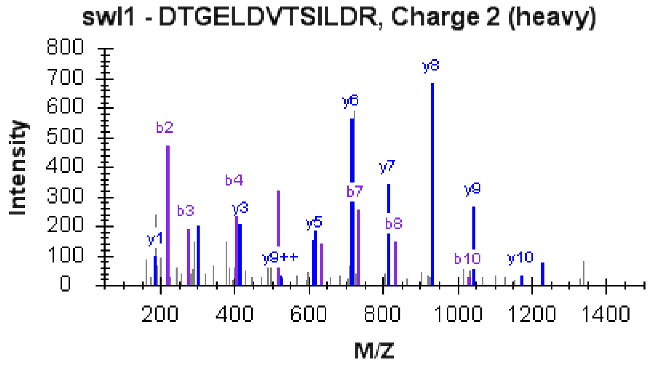

Heavy

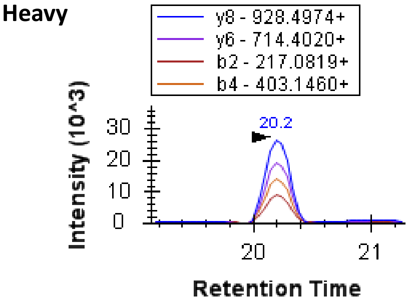

Endogenous

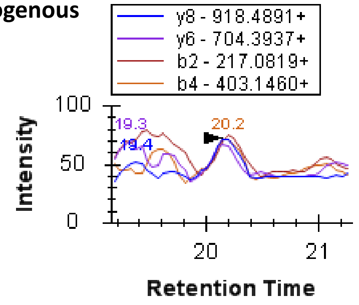

S2A

Interleukin-6 receptor subunit beta

UniProt Entry: P40189
Gene Name: IL6ST

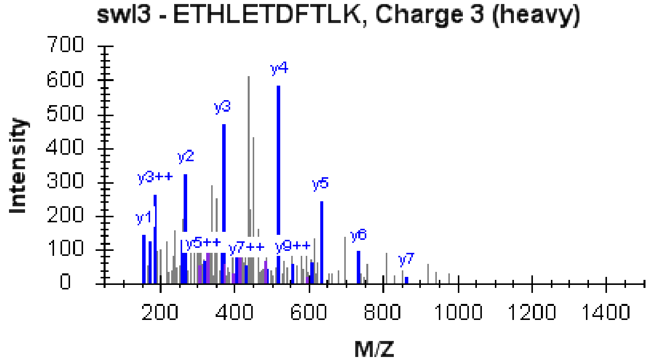

Heavy

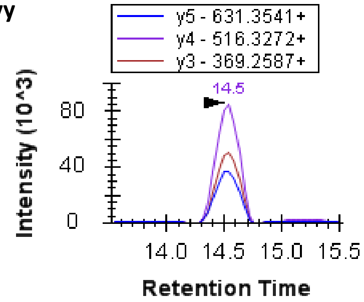

Endogenous

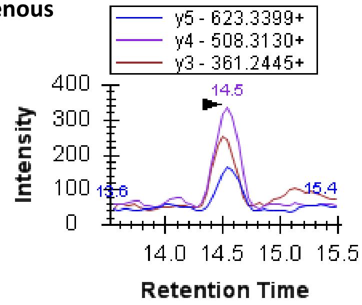

Cell adhesion molecule 1

UniProt Entry: Q9BY67
Gene Name: CADM1

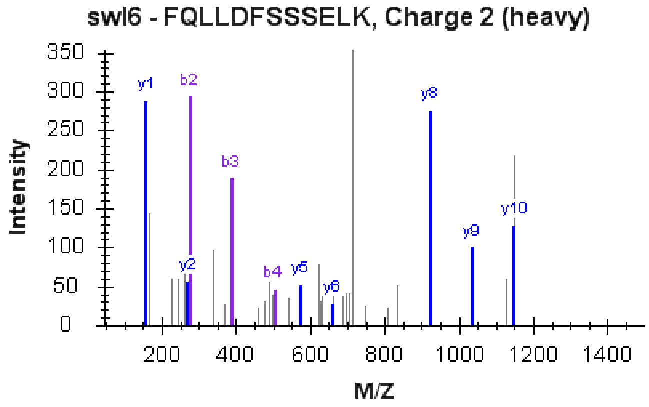

Heavy

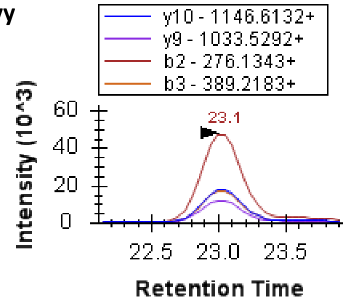

Endogenous

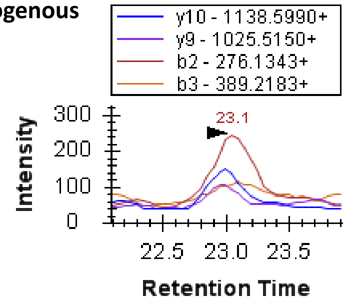

18

S2A

Major prion protein

UniProt Entry: P04156
Gene Name: PRNP

swl1 - GEDFTETDVK, Charge 2 (heavy)

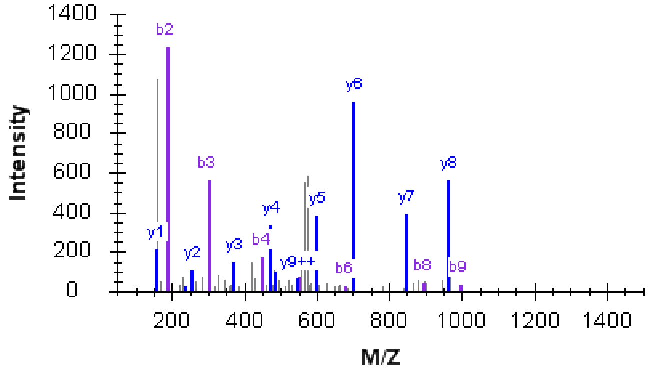

Heavy

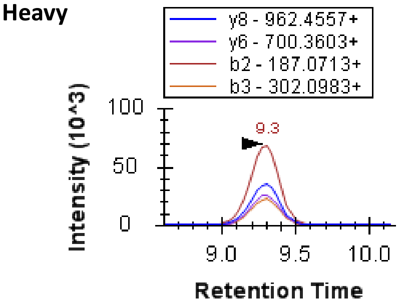

Endogenous

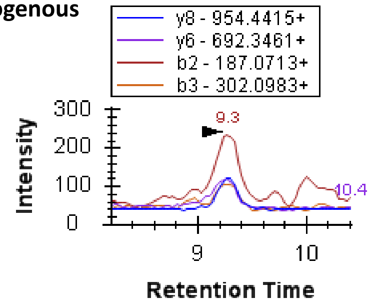

Galectin-3-binding protein

UniProt Entry: Q08380
Gene Name: LGALS3BP

swl10 - GLDLTEDTYKPR, Charge 2 (heavy)

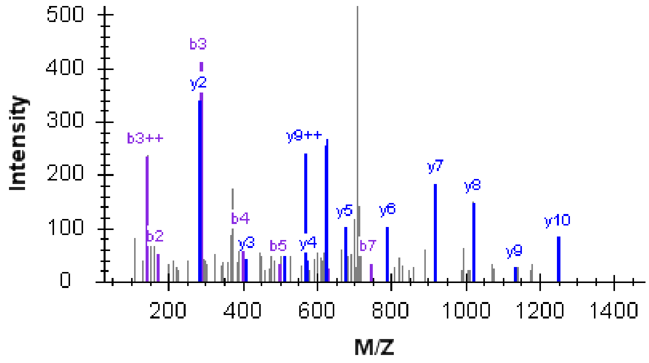

Heavy

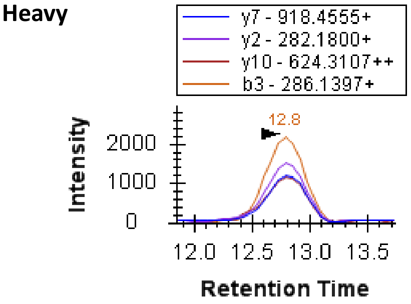

Endogenous

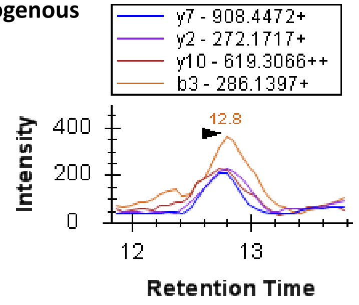

S2A

Serum paraoxonase/arylesterase 1

UniProt Entry: P27169
Gene Name: PON1

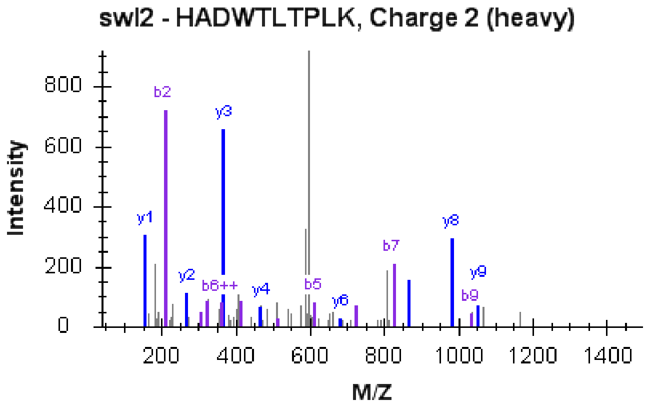

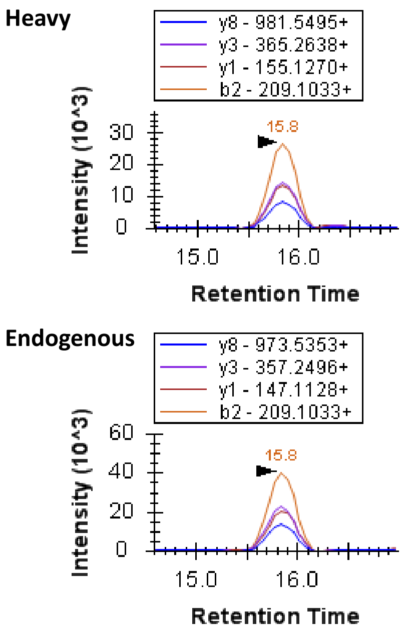

Desmoglein-2

UniProt Entry: Q14126
Gene Name: DSG2

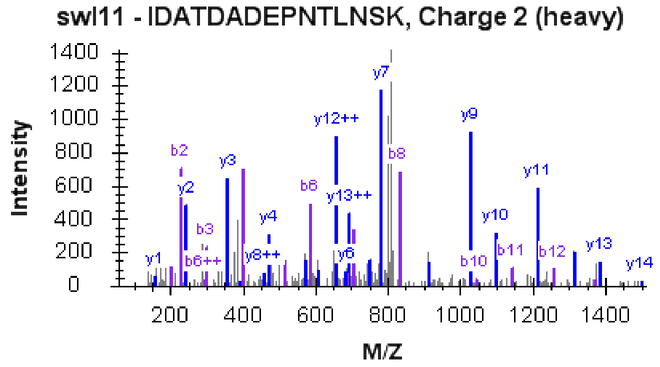

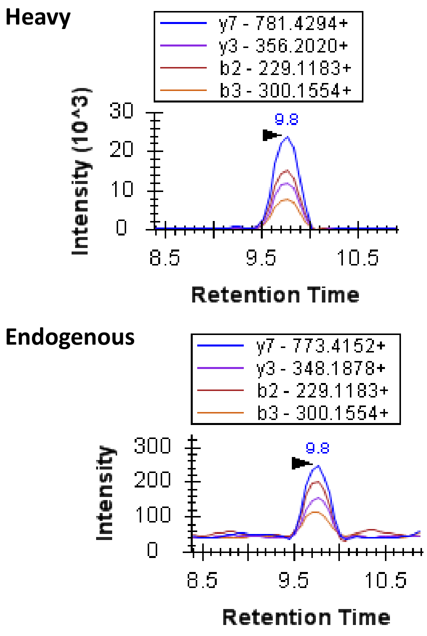

S2A

Attractin

UniProt Entry: O75882
Gene Name: ATRN

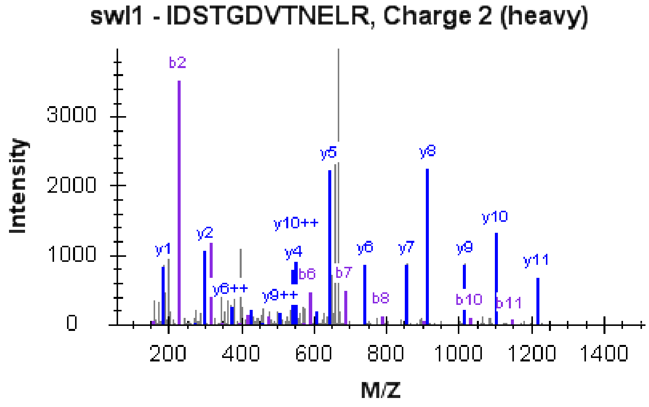

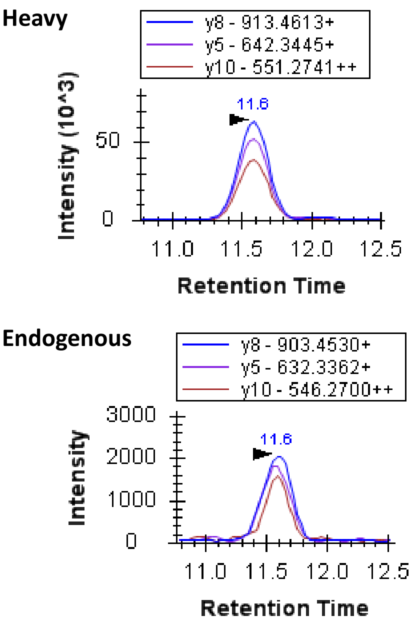

ICOS ligand

UniProt Entry: O75144
Gene Name: ICOSLG

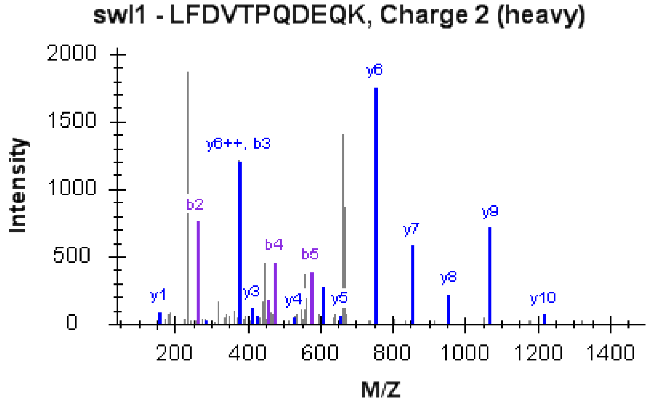

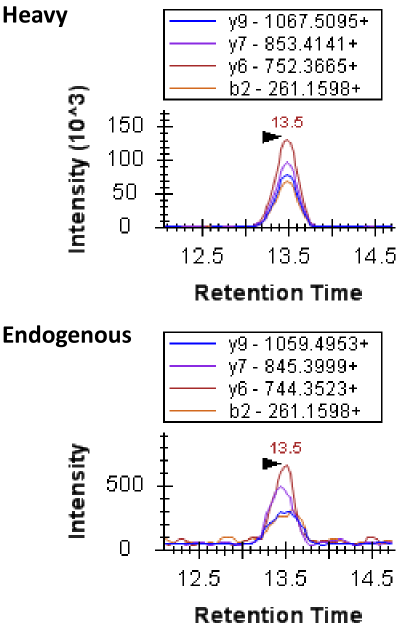

21

S2A

Vasorin

UniProt Entry: Q6EMK4
Gene Name: VASN

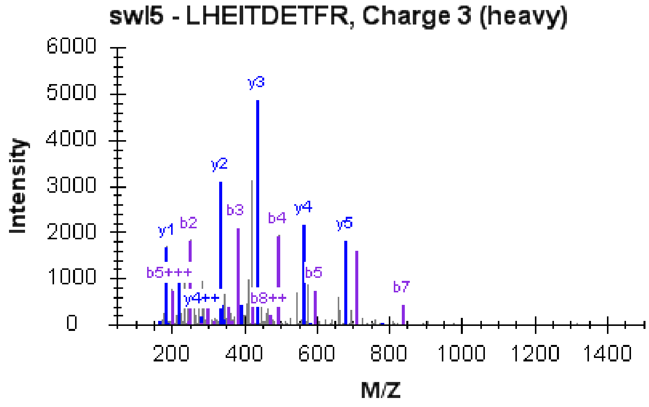

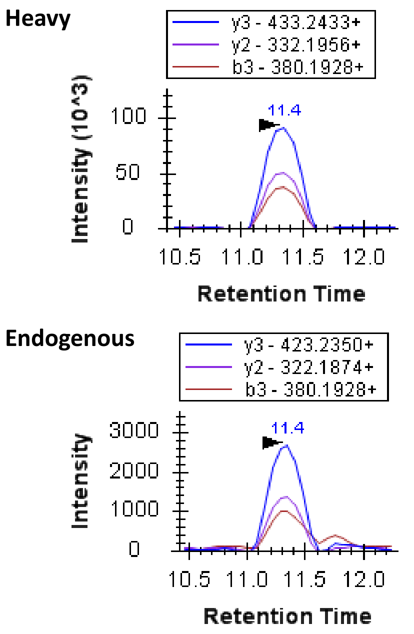

Intercellular adhesion molecule 1

UniProt Entry: P05362
Gene Name: ICAM1

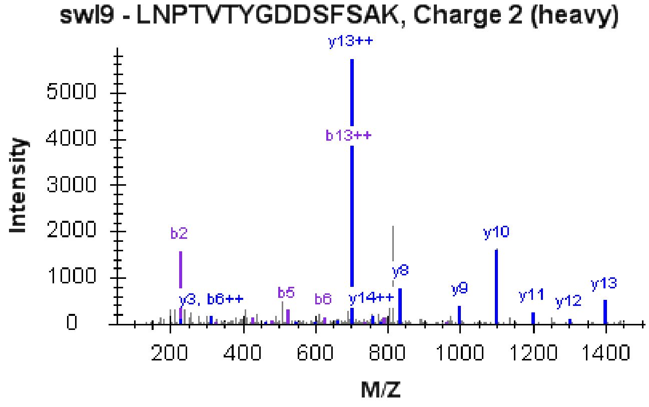

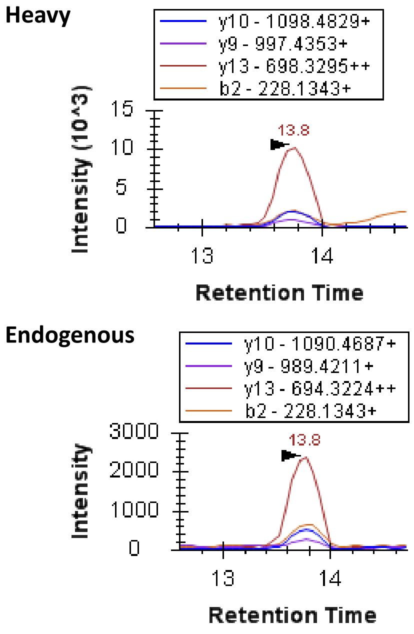

S2A

CD97 antigen

UniProt Entry: P48960
Gene Name: CD97

swl3 - LSAVNSIFLSHDNTK, Charge 3 (heavy)

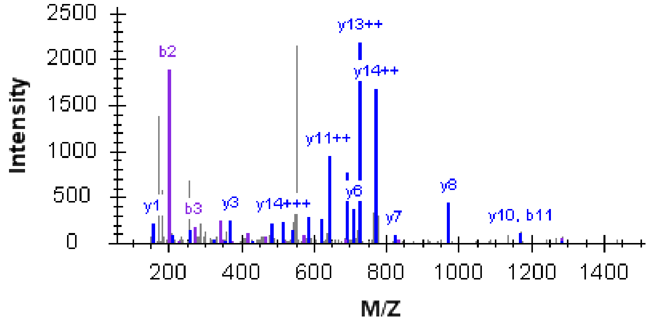

Heavy

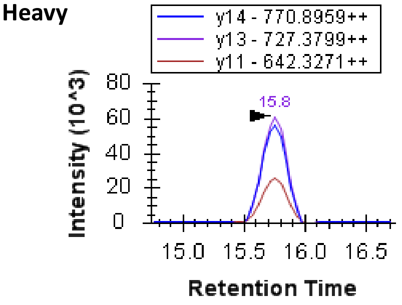

Endogenous

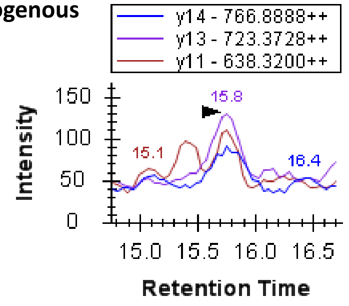

Integrin beta-2

UniProt Entry: P05107
Gene Name: ITGB2

swl9 - LTDNSNQFQTEVGK, Charge 2 (heavy)

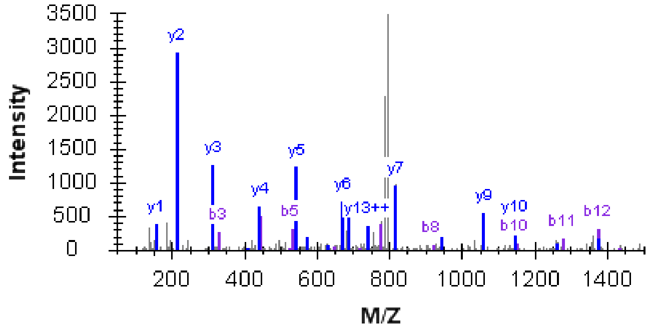

Heavy

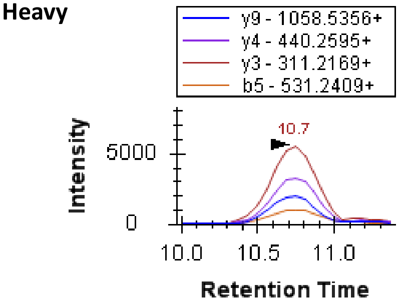

Endogenous

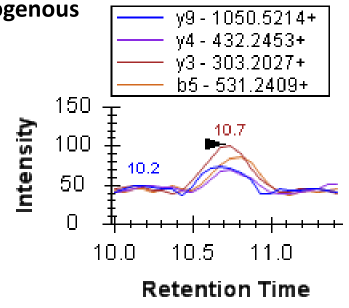

S2A

Macrophage colony-stimulating factor 1

UniProt Entry: P09603
Gene Name: CSF1

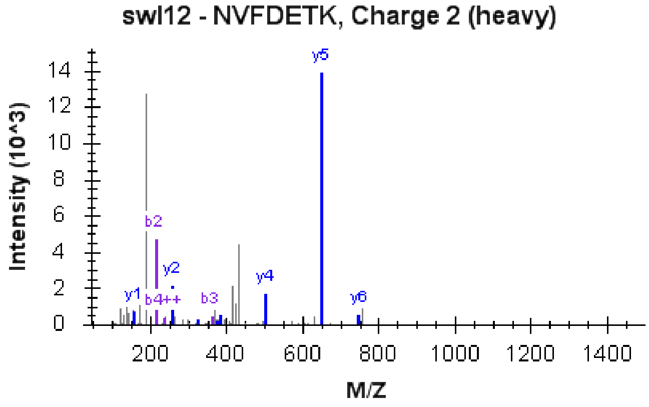

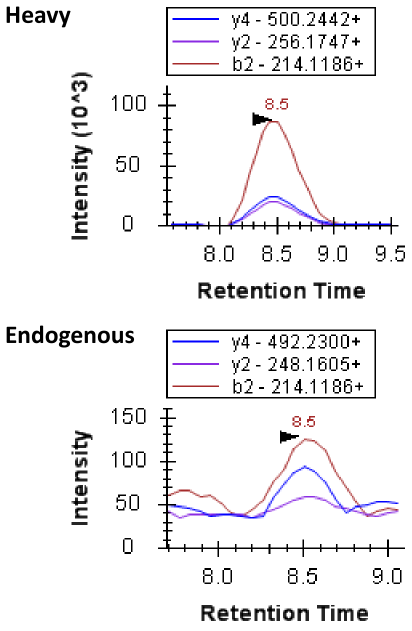

Receptor-type tyrosine-protein phosphatase eta

UniProt Entry: Q12913
Gene Name: PTPRJ

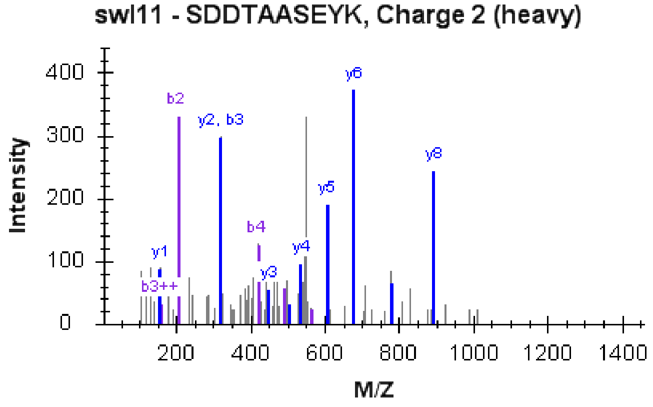

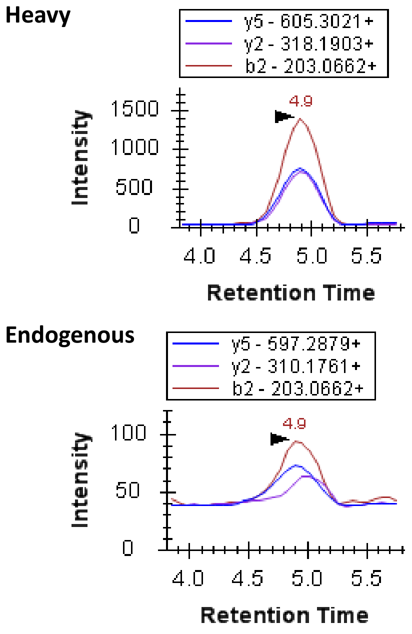

S2A

Interleukin-6 receptor subunit beta

UniProt Entry: P40189
Gene Name: IL6ST

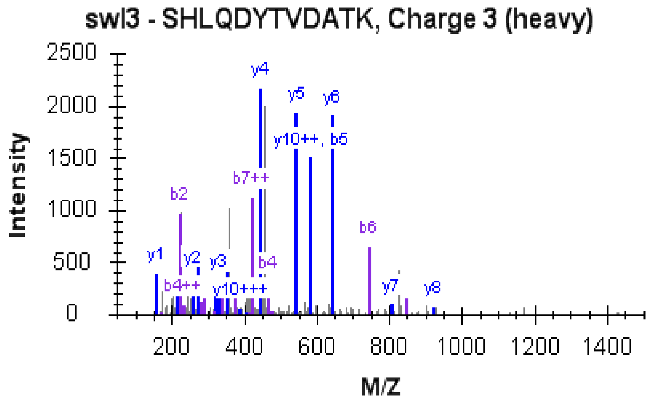

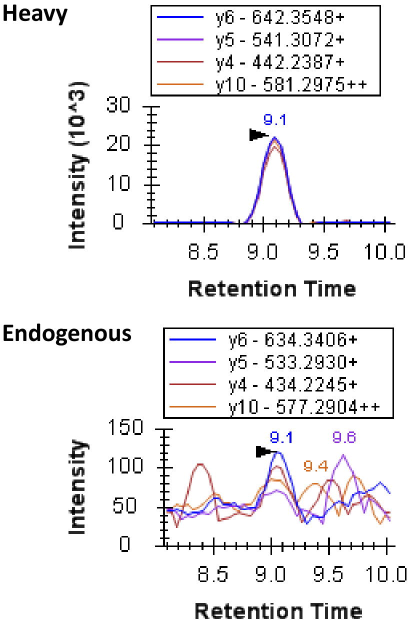

4F2 cell-surface antigen heavy chain

UniProt Entry: P08195
Gene Name: SLC3A2

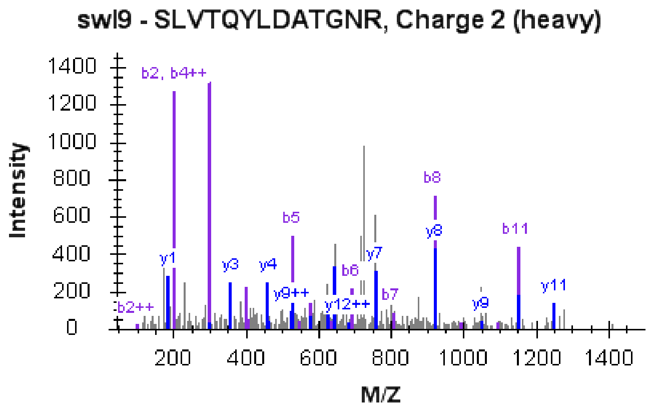

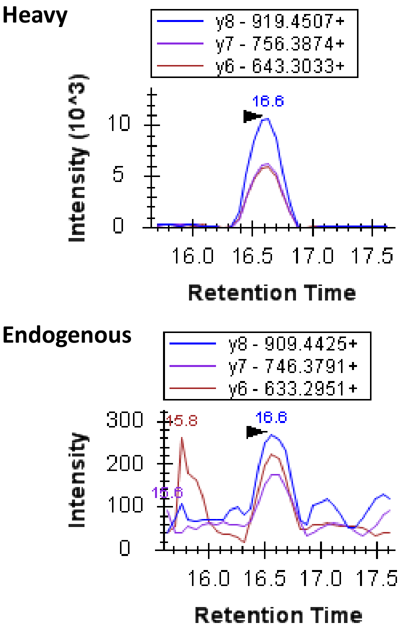

S2A

Hemopexin

UniProt Entry: P02790
Gene Name: HPX

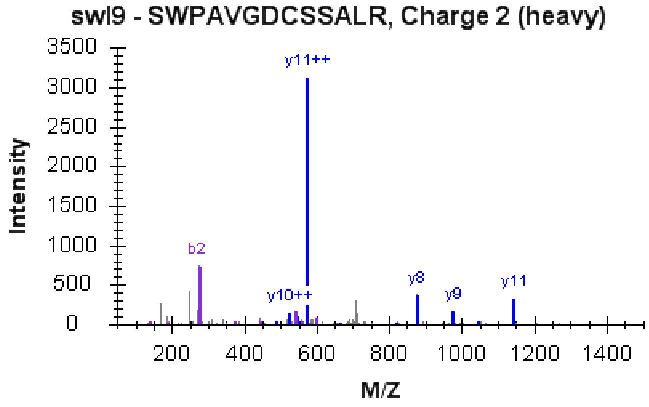

Heavy

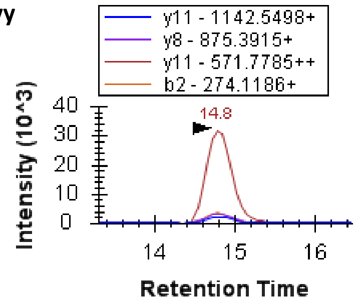

Endogenous

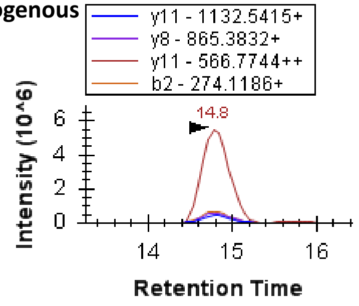

CD276 antigen

UniProt Entry: Q5ZPR3
Gene Name: CD276

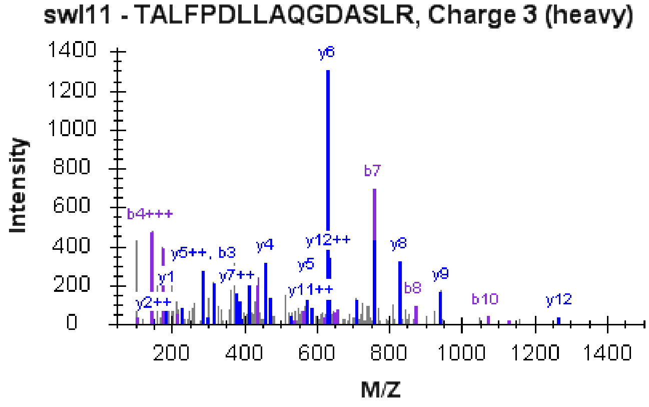

Heavy

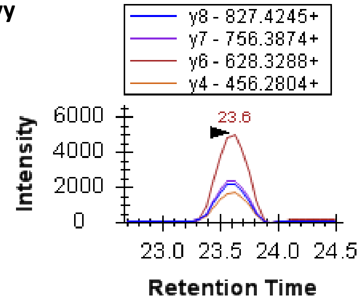

Endogenous

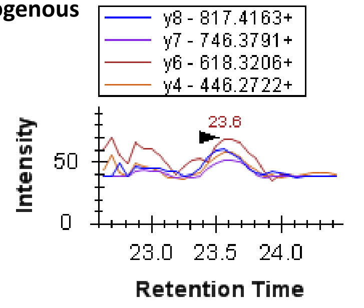

26

S2A

CD109 antigen

UniProt Entry: Q6YHK3
Gene Name: CD109

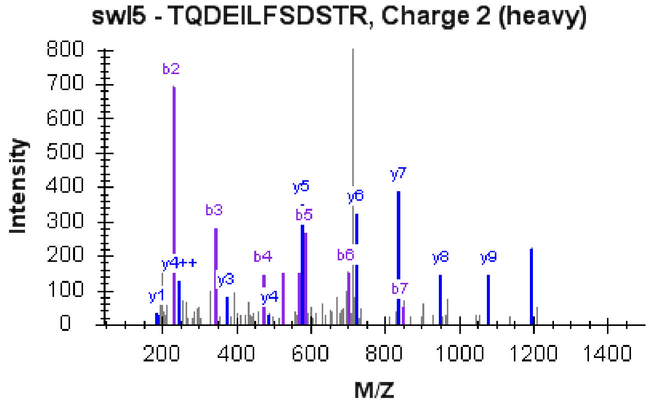

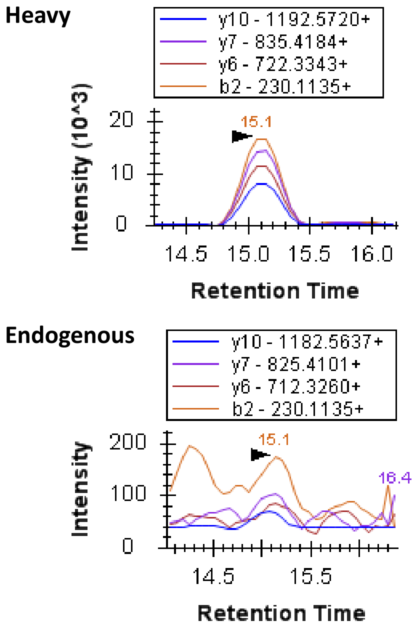

ICOS ligand

UniProt Entry: O75144
Gene Name: ICOSLG

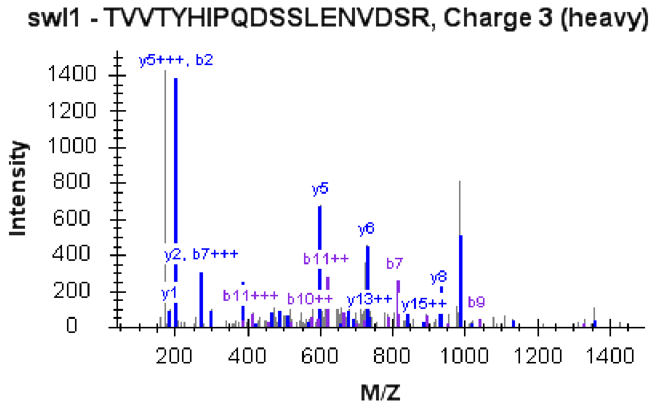

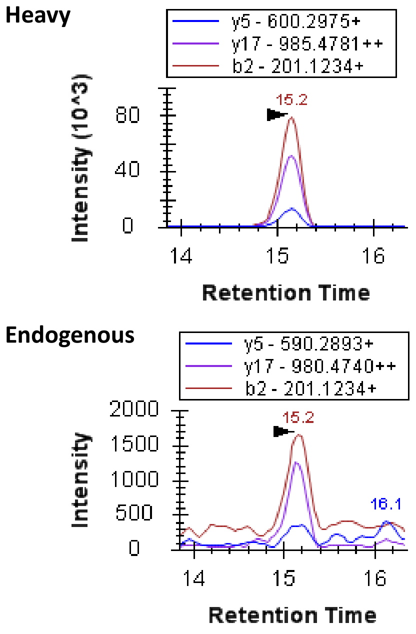

S2A

Hypoxia up-regulated protein 1

UniProt Entry: Q9Y4L1
Gene Name: HYOU1

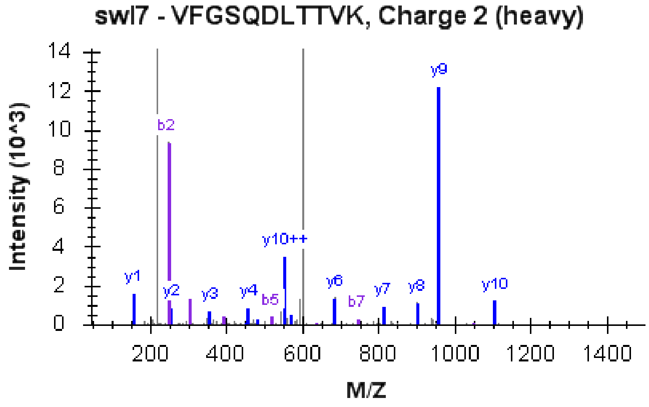

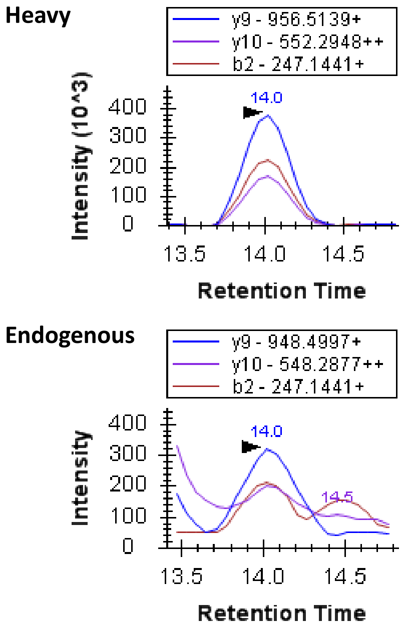

Hypoxia up-regulated protein 1

UniProt Entry: Q9Y4L1
Gene Name: HYOU1

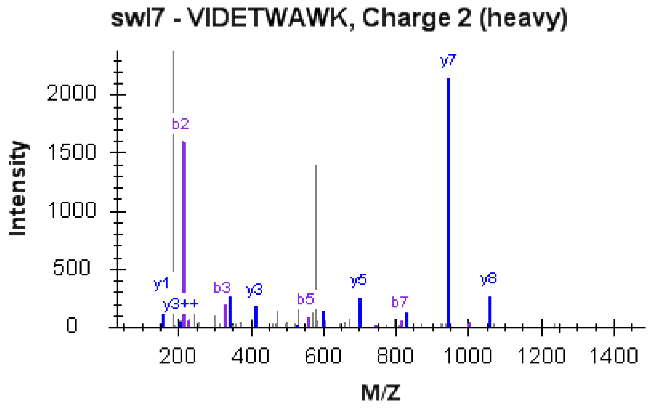

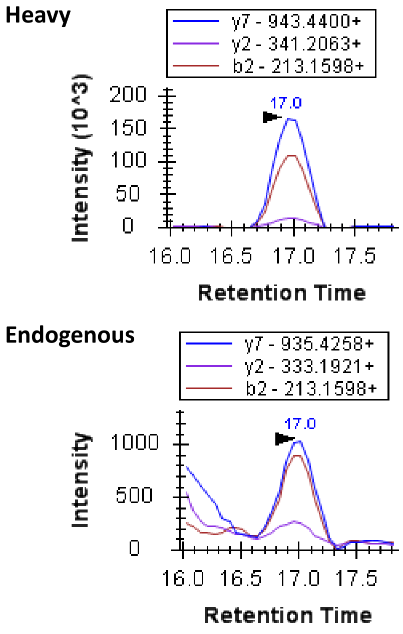

S2A

Receptor-type tyrosine-protein phosphatase eta

UniProt Entry: Q12913
Gene Name: PTPRJ

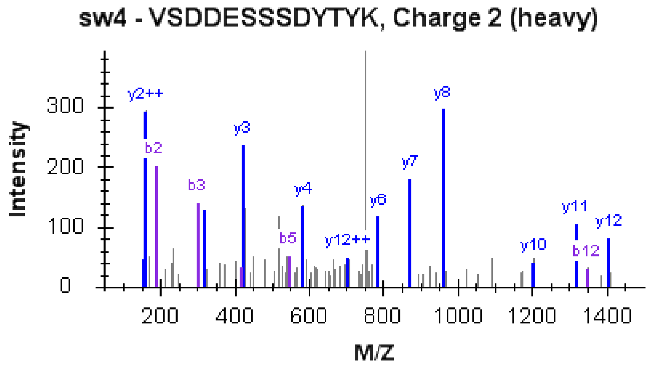

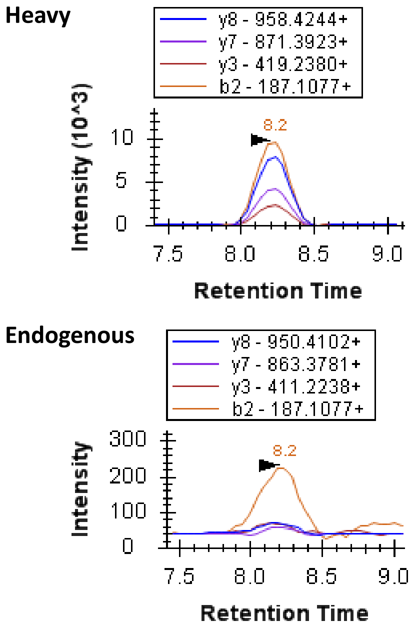

Phospholipid transfer protein

UniProt Entry: P55058
Gene Name: PLTP

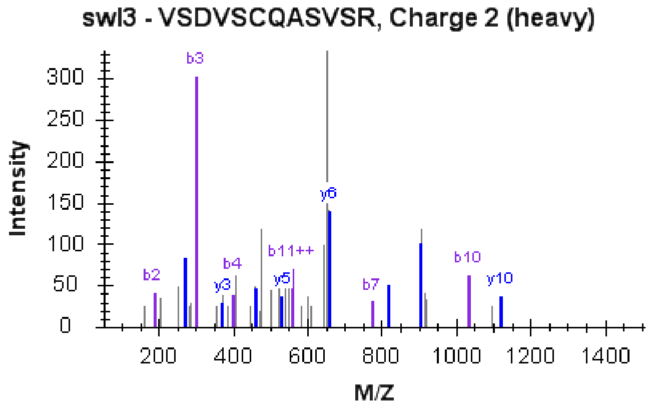

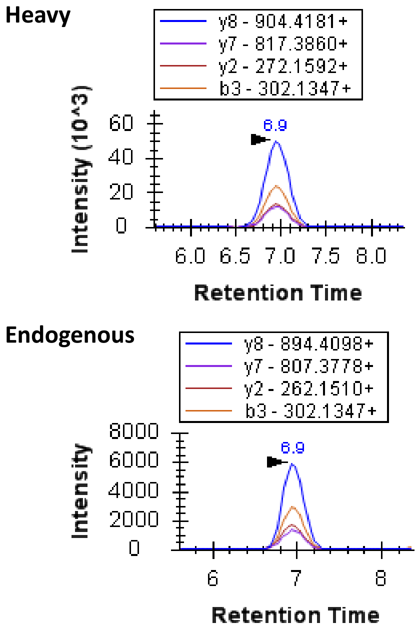

S2A

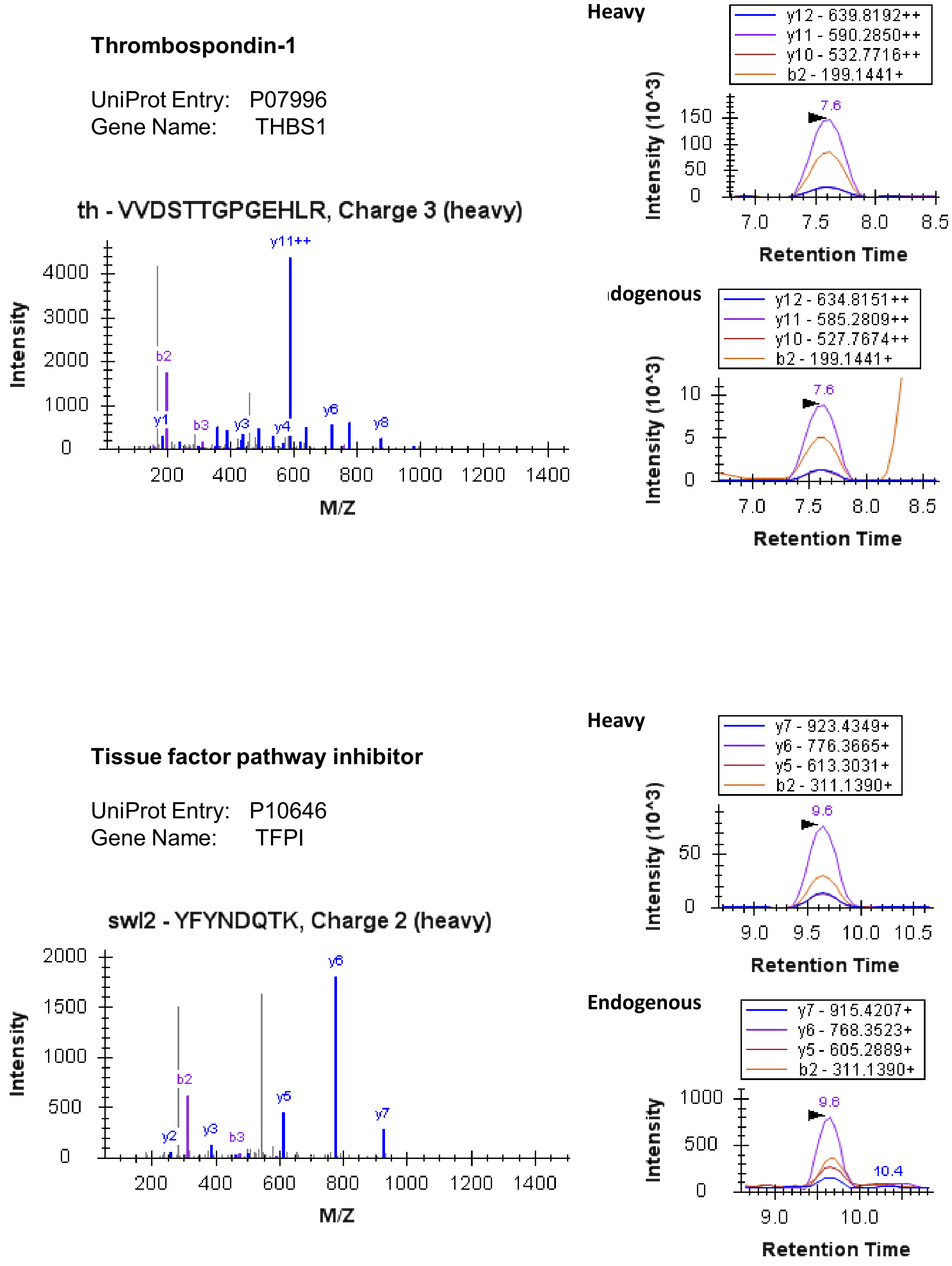
Thrombospondin-1
UniProt Entry: P07996
Gene Name: THBS1
th - VVDSTTGPGEHLR, Charge 3 (heavy)
Intensity
M/Z
y11++
b2
y1
b3
y3
y4
y6
y8
Heavy
y12 - 639.8192++
y11 - 590.2850++
y10 - 532.7716++
b2 - 199.1441+
Intensity (10^3)
Retention Time
7.6
idogenous
y12 - 634.8151++
y11 - 585.2809++
y10 - 527.7674++
b2 - 199.1441+
Tissue factor pathway inhibitor
UniProt Entry: P10646
Gene Name: TFPI
swl2 - YFYNDQTK, Charge 2 (heavy)
y6
b2
y5
y7
y2
y3
b3
Heavy
y7 - 923.4349+
y6 - 776.3665+
y5 - 613.3031+
b2 - 311.1390+
9.6
Endogenous
y7 - 915.4207+
y6 - 768.3523+
y5 - 605.2889+
b2 - 311.1390+
10.4

S2A

Plexin-B1

UniProt Entry: O43157
Gene Name: PLXNB1

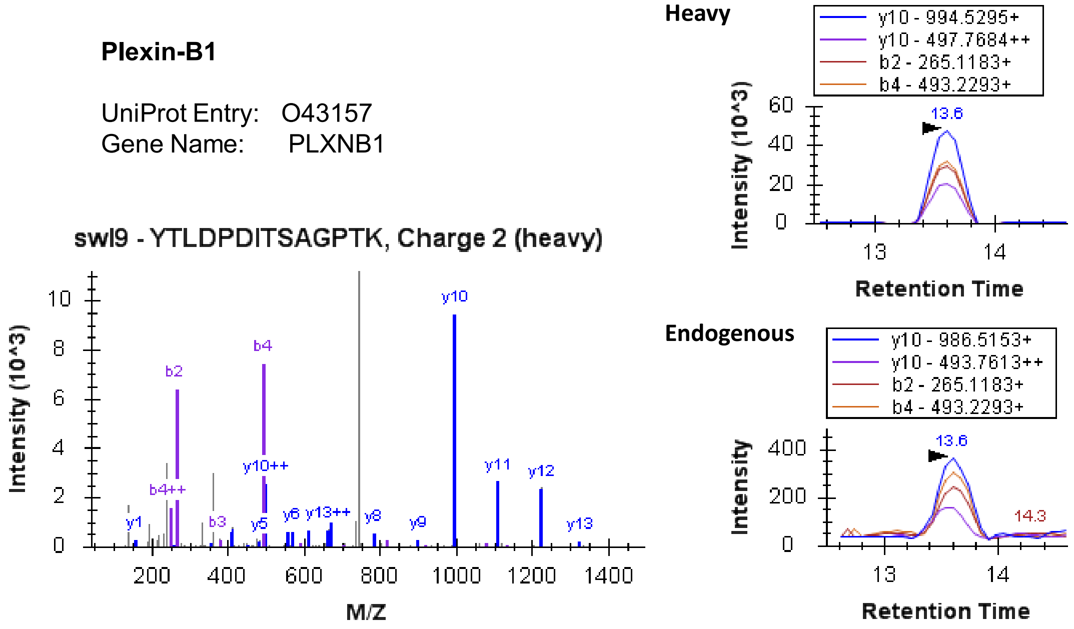

S2B

Tumor necrosis factor receptor superfamily member 3

UniProt Entry: P36941
Gene Name: LTBR

swl3 - AGHFQDTSSPSAR, Charge 3 (heavy)

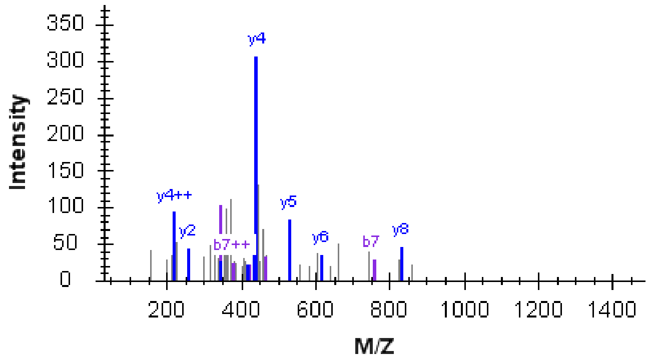

Heavy

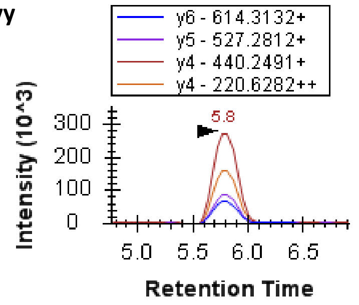

Endogenous

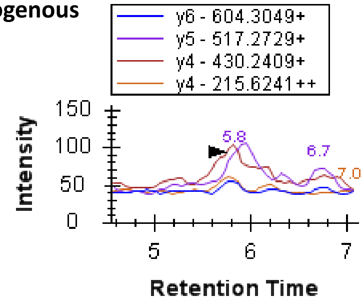

Inactive tyrosine-protein kinase 7

UniProt Entry: Q13308
Gene Name: PTK7

sw4 - DGTPLSDGQSDHTVSSK, Charge 3 (heavy)

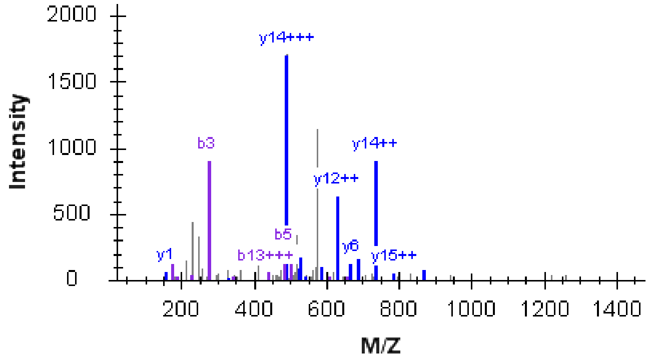

Heavy

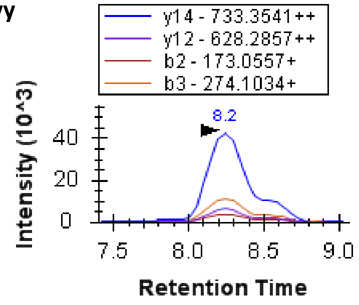

Endogenous

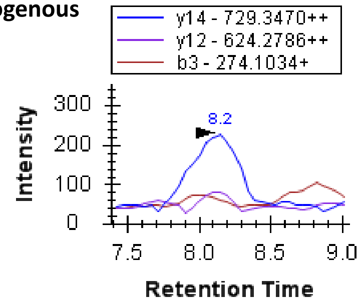

32

S2B

Bone marrow stromal antigen 2

UniProt Entry: Q10589
Gene Name: BST2

Heavy

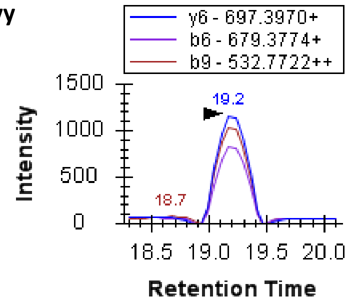

swl11 - DVTHLLQQELTEAQK, Charge 3 (heavy)

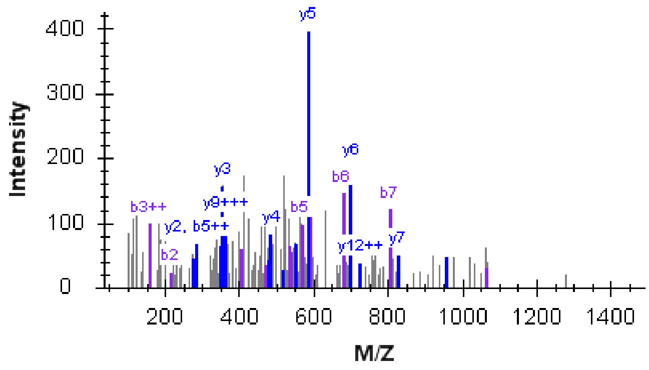

Endogenous

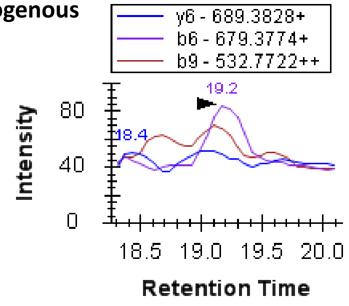

ADAMTS-like protein 4

UniProt Entry: Q6UY14
Gene Name: ADAMTSL4

Heavy

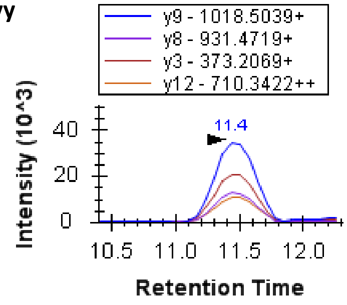

swl5 - EVQCLSTDQTLSTR, Charge 2 (heavy)

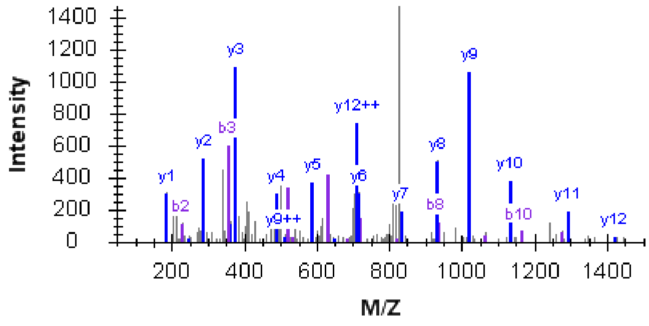

Endogenous

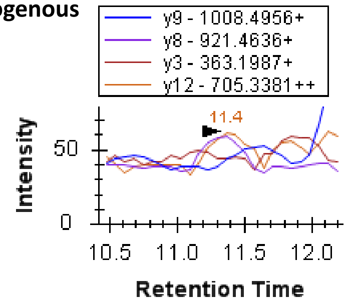

33

S2B

Basigin

UniProt Entry: P35613
Gene Name: BSG

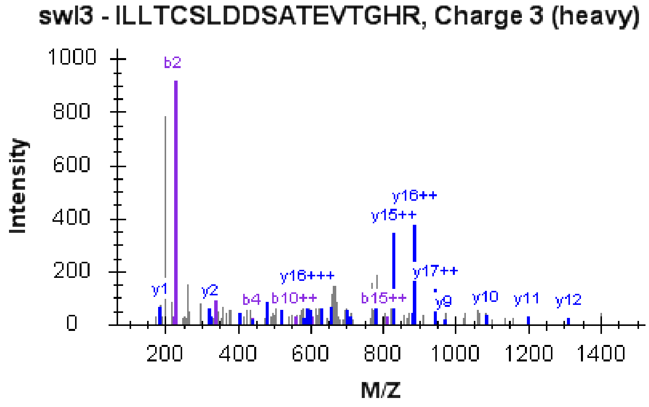

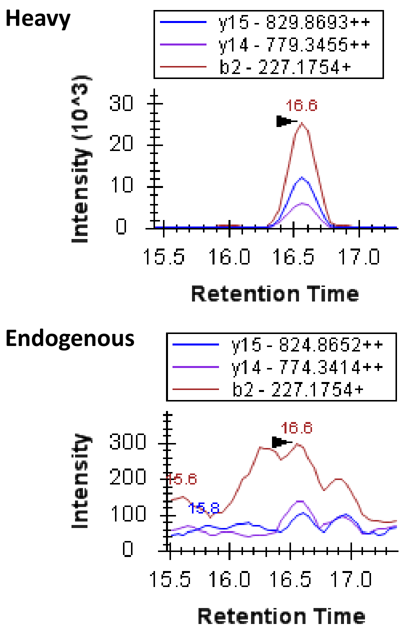

Integrin beta-2

UniProt Entry: P05107
Gene Name: ITGB2

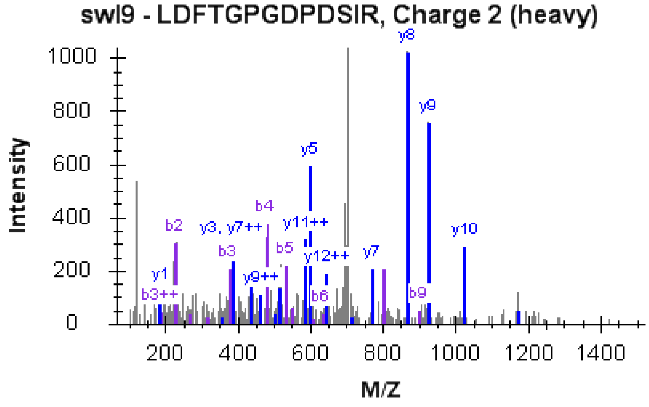

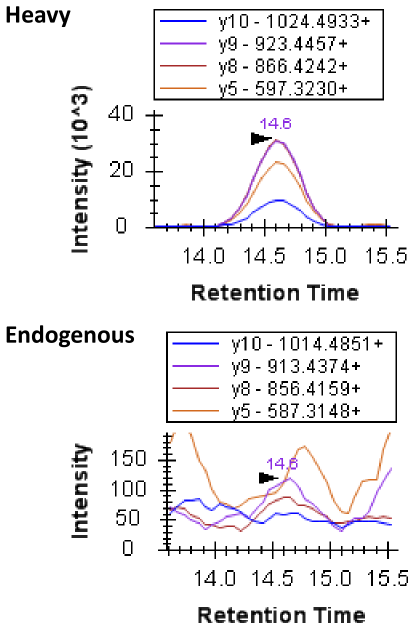

S2B

Teneurin-2

UniProt Entry: Q9NT68
Gene Name: ODZ2

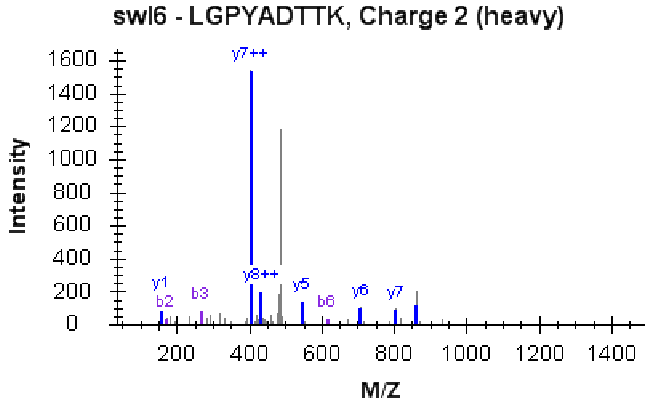

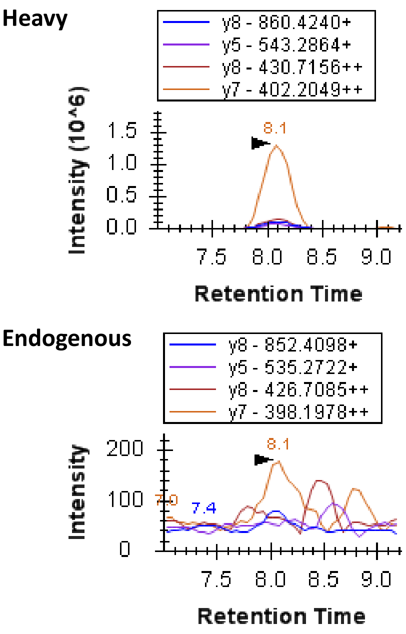

Poliovirus receptor-related protein 1

UniProt Entry: Q15223
Gene Name: PVRL1

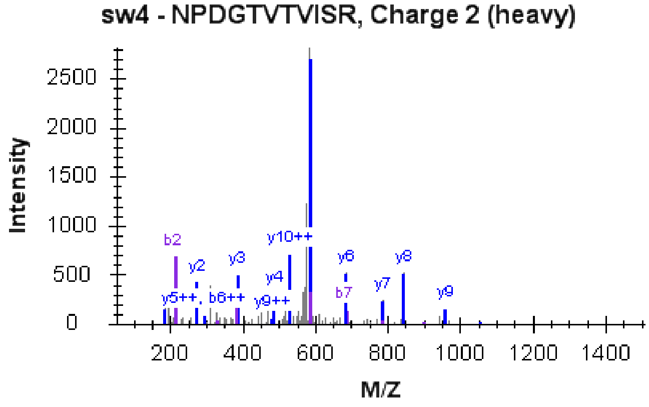

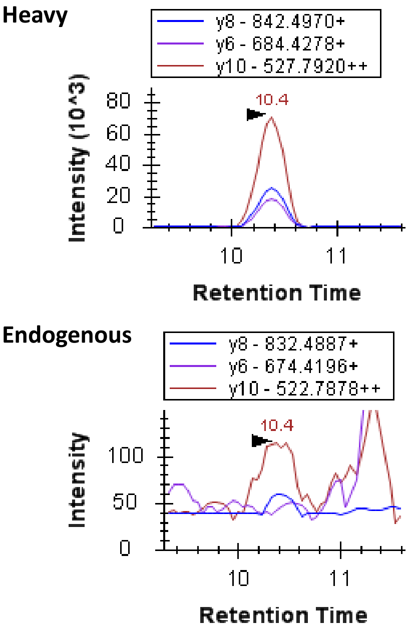

35

S2B

Basal cell adhesion molecule

UniProt Entry: P50895
Gene Name: BCAM

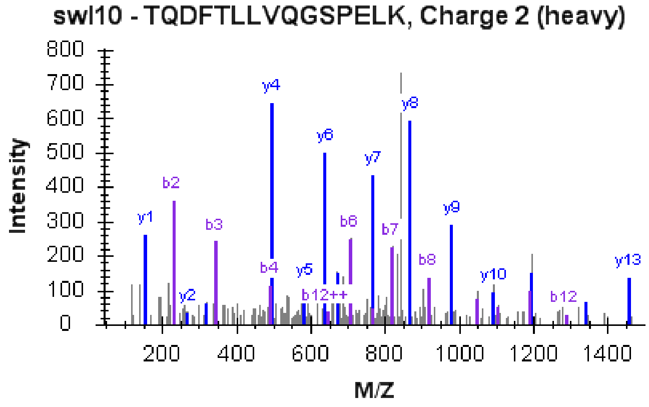

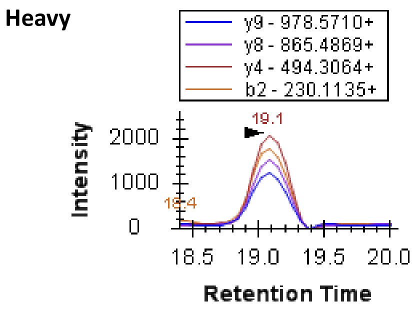

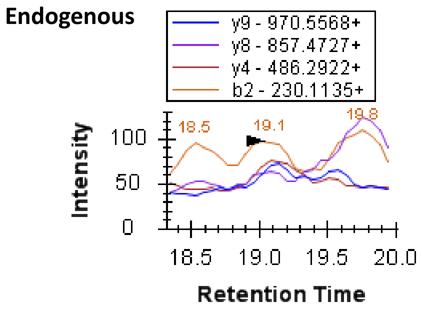

Laminin subunit gamma-1

UniProt Entry: P11047
Gene Name: LAMC1

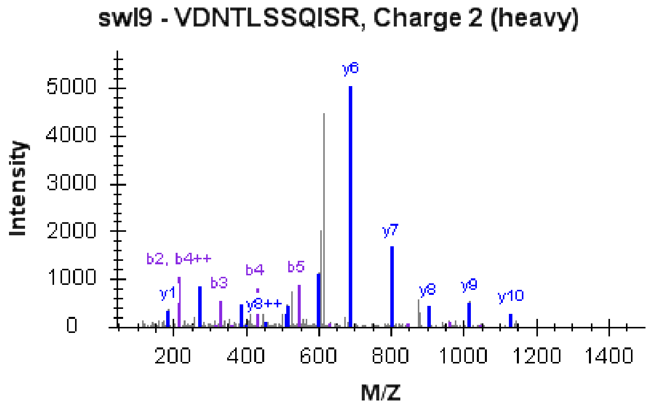

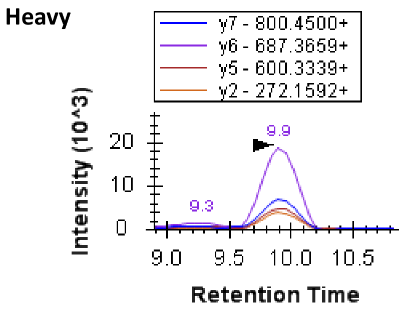

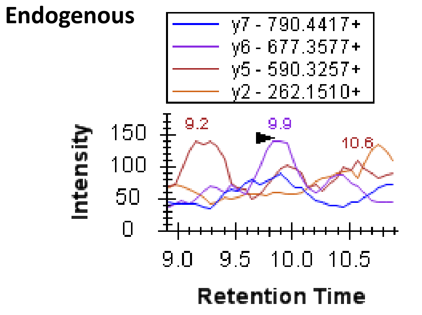

36

S2B

Immunoglobulin superfamily member 3

UniProt Entry: O75054
Gene Name: IGSF3

swl1 - VQGDSTLLHITDLQAR, Charge 3 (heavy)

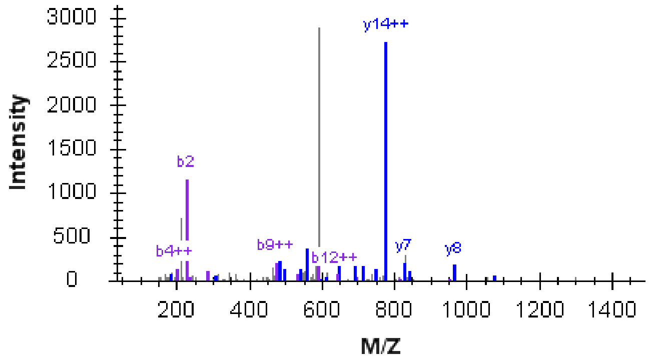

Heavy

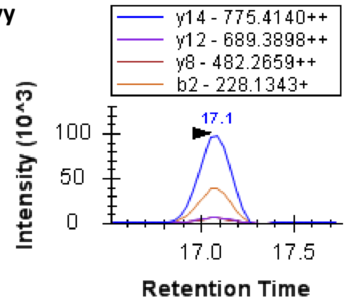

Endogenous

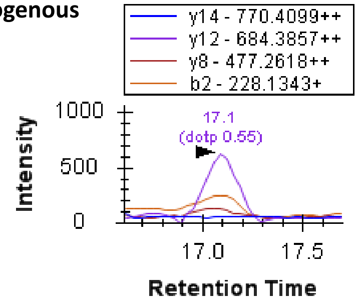

Cell adhesion molecule 1

UniProt Entry: Q9BY67
Gene Name: CADM1

swl11 - VSLTDVSISDEGR, Charge 2 (heavy)

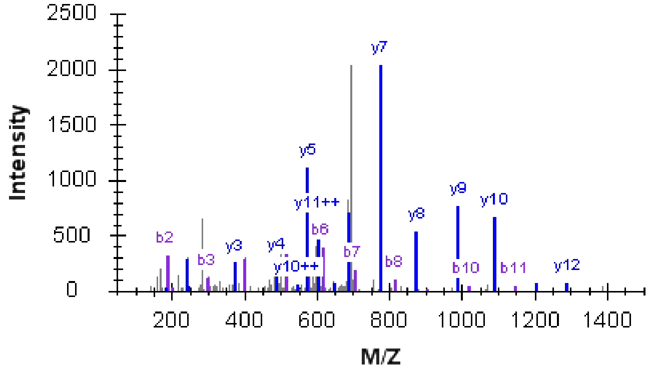

Heavy

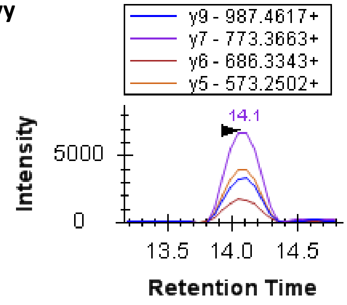

Endogenous

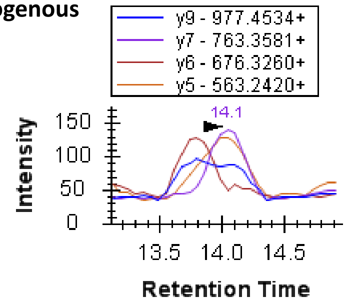

37

S2B

CD276 antigen

UniProt Entry: Q5ZPR3
Gene Name: CD276

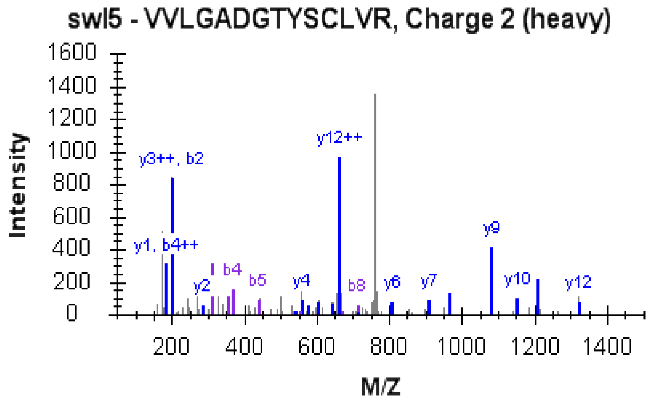

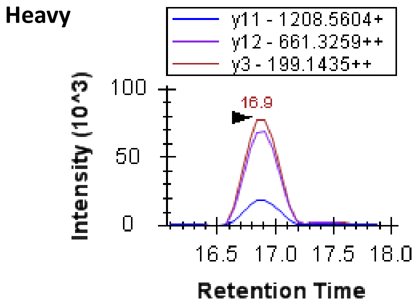

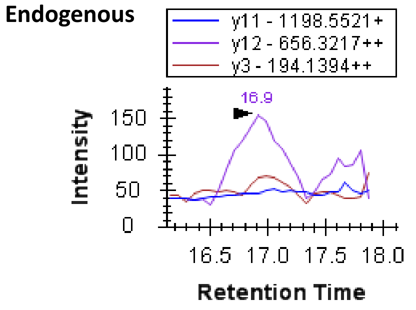

CD97 antigen

UniProt Entry: P48960
Gene Name: CD97

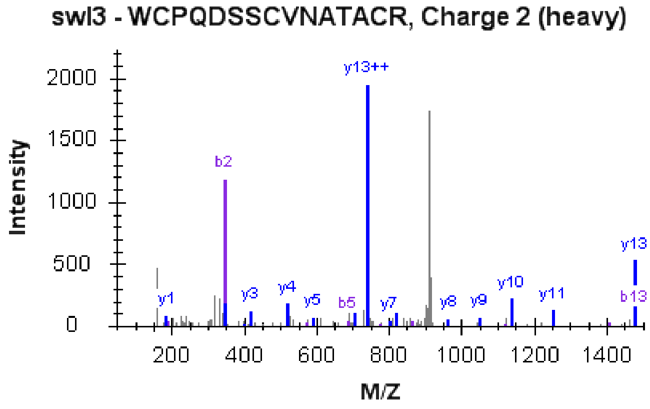

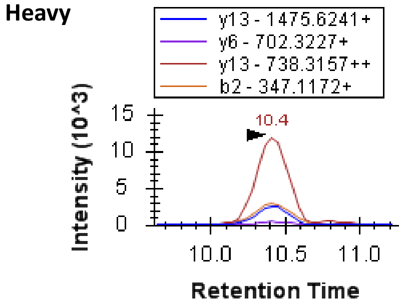

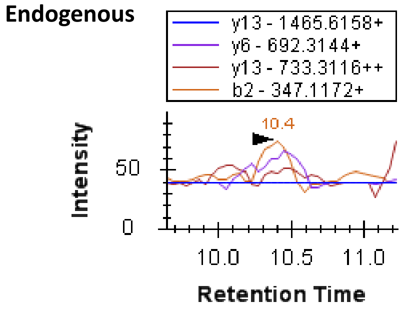

Supplement: Additional file 6: Figure S2 — MPM candidate biomarker peptides detected by SRM in the screening of five MPM sera after enrichment for N-glycopeptides. [file 1559-0275-10-16-S6.pdf]
