# Supplementary material for: Identification of a seven glycopeptide signature for malignant pleural mesothelioma in human serum by selected reaction monitoring
Source: Clin Proteomics. 2013 Nov 8;10(1):16. doi: 10.1186/1559-0275-10-16 (PMC3827840; doi:10.1186/1559-0275-10-16)
Supplement: Additional file 10: Figure S3 — Six glycopeptides panel vs mesothelin ELISA. [file 1559-0275-10-16-S10.doc]

**Additional file 10 to *Cerciello et al.* : Figure S3**

**
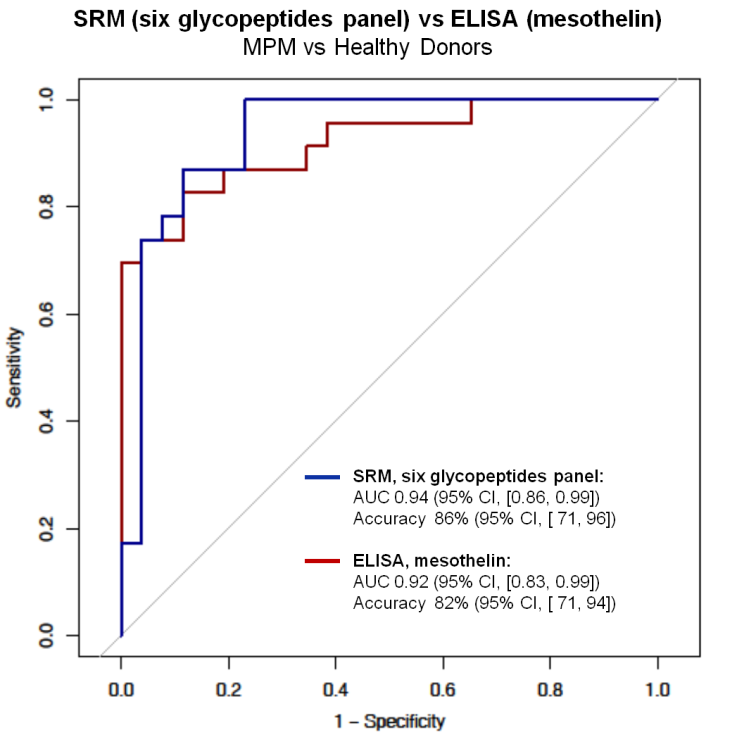
**

**Figure S3. Six glycopeptides panel vs mesothelin ELISA.** Discrimination of 23 MPM subjects from 26 HD using mesothelin ELISA (Mesomark®; red line) or the panel of six glycopeptides (blue line) (**Additional file 3: Table S2)**. AUC indicates area under the curve. Accuracy for MPM vs HD is at cut-off 0.61 and accuracy of mesothelin is at 2 nM cut-off. CI indicates a 95% confidence interval.
