# Supplementary material for: Identification of a seven glycopeptide signature for malignant pleural mesothelioma in human serum by selected reaction monitoring
Source: Clin Proteomics. 2013 Nov 8;10(1):16. doi: 10.1186/1559-0275-10-16 (PMC3827840; doi:10.1186/1559-0275-10-16)
Supplement: Additional file 12 — Supplementary methods. [file 1559-0275-10-16-S12.doc]

**Additional file 12 to *Cerciello et al.* : Supplementary methods**

**Cell culture**

MPM and non-cancerous pleural cell lines were grown in high-glucose Dulbecco modified Eagle medium (DMEM):F12 (Sigma-Aldrich, St Louis, MO) supplemented with 15% fetal calf serum (FCS), 2 mM _L_-glutamine, 1 mM sodium pyruvate, 100 µM β-mercaptoethanol, 0.4 µg/ml hydrocortisone, 10 ng/ml EGF, 10 µg/ml insulin, 5.5 µg/ml transferrin, 6.7 µg/ml selenium, 1% penicillin/streptomycin and nonessential amino acids. ADCA cell lines were maintained with RPMI-1640 (Gibco/Invitrogen) supplemented with 10% FCS, 2 mM _L_-glutamine and 1% penicillin/streptomycin. All cell lines were grown at 37˚C in a 5% CO_2_ humidified atmosphere.

**CSC-based surfaceome analysis and MPM candidate biomarker selection**

General parameters applied for label free relative quantification with the software Superhirn were: MS1 retention time tolerance 1.0 min, MS1 m/z tolerance 10 ppm and MS2 m/z tolerance 30 ppm. Parameters for peptide sequence identification from MS/MS spectra were: mass tolerances of 0.025 Da for precursor and ~ 0.5 Da for fragment ions; trypsin as enzyme for in-silico digestion, with the number of enzyme termini set to 1 and 1 missed cleavage allowed; cysteines with static modification of 57.02146 Da and asparagines with variable modification of 0.98406 Da; probability of peptide and protein identification was assessed using PeptideProphet and ProteinProphet [1, 2] in the transproteomic pipeline (TPP) v4.5 (www. http://tools.proteomecenter.org). For quantification we considered only MS1 features with charge states 2+ to 4+ and assigned to peptide sequences having N- and C-terminal sites of tryptic cleavage and a peptide-mass modification of 0.98406 Da on asparagine-residues after PNGaseF treatment (mass modification after deamidation of the potential site of N-glycosylation and generation of aspartic acid from the former asparagine). Ion-chromatograms of MS1 feature-areas from the same cell line were summed over identical sequences and charge-states, averaged over duplicate runs and considered for quantification if above the noise threshold of 25000 ion counts (for SDM5 only one replicate run was available for quantitative analysis). Log_2_ ratios of MS1 feature-areas of peptides from non-MPM and MPM cell lines [log_2_(ratio_MS1Area_) = log_2_ (non-MPM*_MS1Area_*/MPM*_MS1Area_*)] were calculated pair wise for each MPM and non-MPM cell line for a total of 16 cell lines comparisons. Total intensity normalization was applied for each comparison. A *Z-score* [3] threshold of -0.9 was applied to define features-areas of higher abundance in MPM cell lines and was calculated with the formula : {[log_2_(ratio_MS1Area_) – Mean_log2(ratioMS1Area)_] * (1/Std _log2(ratioMS1Area)_) ≥ (-0.9)}. Higher abundance of peptides was considered reproducible if observed in at least two cell line comparisons (MPM vs non-MPM).

**Spectral libraries of MPM candidate biomarker peptides for SRM-assays generation**

For spectra acquisition, synthetic peptides were resolubilized and mixed in pools of 10 to 20 at final concentration of approximately 150 fmol/µl in 2% ACN, 0.1% FA and analyzed using an Accurate-Mass quadrupole time-of-flight (QTOF) LC/MS series 6520 or 6550 (Agilent Technologies, Santa Clara, CA) equipped with an HPLC-Chip Cube interface (Agilent Technologies). 1 or 2 µl of the peptide mix were loaded from a cooled (6˚C ) micro Well-plate sampler (Agilent Technologies) to an HPLC-chip (5 µm Zorbax C18, enrichment column 160 nL, analytical column 75 µm i.d. x 150 mm length; Agilent Technologies) using a capillary-pump at 1.5 µl/min flow rate (Agilent Technologies). After automated switch of the HPLC-Chip system to analysis mode, peptides were eluted with a linear gradient of 5 to 35% ACN/water, 0.1% FA over 30 or 70 min using a nano-pump at flow rate of 300 nl/min. (Agilent Technologies). The MS was operated in a data dependent acquisition mode of 1 MS scan followed by 5 MS/MS scans with absolute precursor threshold of 800 ion counts and scan speed based on precursor abundance (target 25000 counts/spectrum). A selection of maximal 5 precursor ions with 2+ or 3+ or unknown charge state per cycle was allowed with active exclusion after 2 spectra released after 1 min. Cycle time was set to 4 s with acquisition rate of 2.75 MS spectra and 1.4 MS/MS spectra per second. Mass ranges for MS and MS/MS were between 100 respectively 300 m/z and 1700 m/z. Collision energy (CE) was calculated with the formula [(3.6 * (m/z)/100) – 4.8]. After conversion of raw data in mzXML format [4], the software Sorcerer2^TM^-Sequest (Sage-N Research, Milpitas, CA) was used to search MS/MS spectra against a database where the concatenated sequences of the synthesized MPM candidate biomarker peptide were integrated as a virtual single protein in the background of the human UniprotSwissprot database v56.9. Search parameters were set as following: enzyme trypsin and fully tryptic termini required, peptide mass tolerance of 50 ppm, static modifications of 57.021465 Da for cysteines and variable modifications of 8.014199 Da for heavy lysines and 10.008269 for heavy arginines.

**Generation and optimization of SRM-assays**

For initial SRM-assays, precursor peptide-ions at charge states 2+, 3+ or both if available were selected. Fragment-ions from the series y+, y++ and b+, b++ were considered. Six transitions per peptide and charge state were manually selected based on optimal signal intensities of heavy isotope-labeled peptides spiked (SpikeTides_L™, JPT Peptide Technologies) in the matrix of serum enriched for N- glycopeptides. Formula for the CE energy was identical to that used for the generation of the spectral libraries (CE = [(3.6 * (m/z)/100) – 4.8]). Retention time was empirically assessed based on the elution time of the heavy isotope-labeled peptides (SpikeTides_L™, JPT Peptide Technologies). Skyline was used to calculate the corresponding light forms of the transitions. For endogenous peptides detected in serum, further assay optimization was performed manually using heavy isotope-labeled peptides (SpikeTides_L™, JPT Peptide Technologies) spiked in the matrix of serum enriched for N-glycopeptides. One precursor charge state per peptide (2+ or 3+) was selected based on SRM signal intensities of the light (endogenous) form in serum. Four fragment-ions per precursor charge state were selected based on signal intensity and at least three transitions per peptides were defined as reference quantifier for quantification. CEs were individually optimized for each transition using the software Skyline [5]. Starting from the CE calculated by the formula [(3.6 * (m/z)/100) – 4.8], CE was stepwise increased or decreased by 1 V for a total of 11 steps and best CE energy was selected based on optimal signal intensity as manually assessed.

**Serum samples**

Peripheral whole blood was collected by needle venipuncture in serum tubes and allowed to clot for at least 30 minutes. After centrifugation at 1620 x g for 10 min, serum was separated from the clotted samples, supplemented with 10 mM EDTA and centrifuged again at 1620 x g for 10 min before aliquoting and storing at -80˚C.

**Serum enrichment for N-glycopeptides and MS analysis**

For the enrichment of N-glycopeptides, proteins from 100 µl of serum were denatured in 300 µl of 8 M urea, 0.2 M Tris-HCl buffer, pH 8.5, reduced with 4.5 mM dithiothreitol (DTT) for 1 h at 37˚C with gentle shaking and alkylated in the dark with 23 mM of iodoacetamide for 30 min at room temperature with gentle shaking. For tryptic digestion, urea concentration was reduced to 2 M by diluting with 0.2 M Tris-HCl buffer, pH 8.5, and 40 µg of trypsin (Sequencing Grade Modified Trypsin, Promega, Madison, WI) per sample were added followed by 16 h incubation at 37˚C with gentle shaking. Peptides were than desalted and concentrated using Sep-Pak C18 cartridges (Waters, Milford, MA) and oxidized for 1 h with 8 mM sodium meta-periodate in 20 mM sodium acetate, 100 mM sodium chloride, pH 5.0, at 6˚C in the dark and with gentle shaking. Peptides were desalted and concentrated again and coupled overnight to Affi-Prep Hz hydrazide resin beads (Bio-Rad, Hercules, CA) with gentle head-over rotation at room temperature. To remove non-glycosylated peptides, beads were washed 10x with 5 M sodium chloride, 5x with water, 10x with 80% ACN /20% water, 10x with methanol, 10x with water and finally 10x with 100 mM disodium-hydrogenphosphate buffer, 25 mM EDTA, pH 7.1. N-glycopeptides were then released from the beads by overnight incubation with 1 µl of the enzyme PNGaseF (500 U/µl) (New England Biolabs, Ipswich, MA) per sample at 37˚C and gentle head-over rotation. Peptides were desalted by Sep-Pak C18 cartridges, dried and finally resuspended in 40 µl of 2% ACN, 0.1% FA before MS analysis.

Analysis of serum samples enriched for N-glycopeptides was performed on a triple quadrupole LC/MS 6460 series (Agilent Technologies) equipped with an HPLC-Chip Cube interface (Agilent technologies) and operating in positive ion SRM mode. For chromatography, an HPLC system series 1200 (Agilent Technologies) was used connected to a large capacity HPLC-Chip (5 µm Zorbax C18, enrichment column 160 nl, analytical column 75 µm i.d. x 150 mm length). 1.5 µl of the peptide mixture were loaded for 2 min. from the cooled (6˚C) micro Well-plate sampler (Agilent Technologies) to the enrichment column of the HPLC-Chip using a capillary-pump at flow rate of 2 µl/min. Peptides were then separated in the analytical column using a nano-pump at flow rate of 300 nl/min and a linear gradient of 5 to 35% ACN/water, 0.1 % FA over 30 min. After peptide separation, ACN was increased from 35 to 95% in 2 min and the column washed at 95% ACN for 6 min followed by a re-equilibration phase at 5% ACN for 6.5 min. Capillary voltage for electrospray ionization was set between 1870 and 1950 V, fragmentor voltage at 130 V and cell accelerator voltage at 4 V. Energy for collision induced dissociation was calculated with the formula [(3.6 * (m/z)/100) – 4.8] or an optimized value was used. Resolution of Q1 and Q3 was set to unite corresponding to 0.7 FWHM (full width, half maximum). Data were acquired with the software MassHunter Workstation Acquisition vB.04.01 (Agilent Technologies) and raw Agilent.d files imported in the software Skyline.

**References**

1. Keller A, Nesvizhskii AI, Kolker E, Aebersold R: **Empirical statistical model to estimate the accuracy of peptide identifications made by MS/MS and database search.** *Anal Chem* 2002, **74:**5383-5392.

2. Nesvizhskii AI, Keller A, Kolker E, Aebersold R: **A statistical model for identifying proteins by tandem mass spectrometry.** *Anal Chem* 2003, **75:**4646-4658.

3. Quackenbush J: **Microarray data normalization and transformation.** *Nat Genet* 2002, **32 Suppl:**496-501.

4. Pedrioli PG, Eng JK, Hubley R, Vogelzang M, Deutsch EW, Raught B, Pratt B, Nilsson E, Angeletti RH, Apweiler R, et al: **A common open representation of mass spectrometry data and its application to proteomics research.** *Nat Biotechnol* 2004, **22:**1459-1466.

5. Maclean B, Tomazela DM, Abbatiello SE, Zhang S, Whiteaker JR, Paulovich AG, Carr SA, Maccoss MJ: **Effect of collision energy optimization on the measurement of peptides by selected reaction monitoring (SRM) mass spectrometry.** *Anal Chem* 2010, **82:**10116-10124.
